# Unique residues in the ATP gated human P2X7 receptor define a novel allosteric binding pocket for the selective antagonist AZ10606120

**DOI:** 10.1038/s41598-017-00732-5

**Published:** 2017-04-07

**Authors:** Rebecca C. Allsopp, Sudad Dayl, Ralf Schmid, Richard J. Evans

**Affiliations:** 1 Department of Molecular and Cell Biology, University of Leicester, Leicester, UK; 2 Department of Chemistry, College of Science, University of Baghdad, Baghdad, Iraq; 3 Leicester Institute of Structural and Chemical Biology, University of Leicester, Leicester, UK

## Abstract

The P2X7 receptor (P2X7R) for ATP is a therapeutic target for pathophysiological states including inflammation, pain management and epilepsy. This is facilitated by the predicted low side effect profile as the high concentrations of ATP required to activate the receptor are usually only found following cell damage/disease and so P2X7Rs respond to a “danger” signal and are not normally active. AZ10606120 is a selective antagonist for P2X7Rs (IC_50_ of ~10 nM) and ineffective at the P2X1R (at 10 μM). To determine the molecular basis of selectivity we generated a series of P2X7/1R chimeras and mutants. Two regions that are unique to the P2X7R, a loop insertion (residues 73–79) and threonine residues T90 and T94, are required for high affinity antagonist action. Point mutations ruled out an orthosteric antagonist site. Mutations and molecular modelling identified an allosteric binding site that forms at the subunit interface at the apex of the receptor. Molecular dynamics simulations indicated that unique P2X7R features regulate access of AZ10606120 to the allosteric site. The characterisation of the allosteric pocket provides a new and novel target for rational P2X7R drug development.

## Introduction

The P2X7 receptor (P2X7R) is a cation channel opened by the binding of extracellular ATP^1^, expressed on a range of cell types, and has an ATP EC_50_ of ~0.3–1 mM at physiological concentrations of calcium and magnesium^2^. This EC_50_ is considerably higher than that required for other P2XR subtypes (~13 distinct recombinant receptor phenotypes formed from the homo- and heterotrimeric assembly of the P2X1-7 subunits that constitute a structurally distinct family of ligand gated ion channels)^2^. Such high levels of ATP are not usually found in healthy tissues and P2X7Rs therefore generally have negligible activity under normal conditions. However at sites of inflammation, cellular damage, necrosis and phagocytic degranulation extracellular ATP levels can rise to ~mM levels activating P2X7Rs; the receptor can therefore be considered to respond to “danger” signals. The binding of ATP to the P2X7R opens the channel pore and allows flux of cations. On prolonged stimulation (>10 s) the passage of larger molecules (up to 900 Da e.g. the fluorescent dyes ethidium and YO-PRO) can be detected^3^, and sustained activation eventually leads to cell death^4^. In macrophages, P2X7R stimulation activates the inflammasome, IL-1β secretion, and an immune response^5^. P2X7Rs are also expressed by other cell types including neurons, astrocytes, oligodendrocytes, osteoblasts, fibroblasts, endothelial and epithelial cells^6^. Animal studies have shown that reduction in P2X7R receptor activity (genetic manipulation or antagonists) can alleviate a range of conditions including inflammatory and neuropathic pain, epilepsy, neurodegenerative diseases and transplant rejection^6^. In humans single nucleotide P2X7R polymorphisms have been associated with several conditions including pain sensitivity^7^, bipolar disorder and depression^8^. P2X7R selective antagonists therefore have considerable therapeutic potential in a range of disease states.

Drug library screening identified *N*-[2-[[2-[(2-Hydroxyethyl)amino]ethyl]amino]-5-quinolinyl]-2-tricyclo[3.3.1.13,7]dec-1-ylacetamide dihydrochloride, known as AZ10606120 as a selective, high affinity antagonist at human and rat P2X7Rs, with little or no effect at other P2XR subtypes^9, 10^. In animal studies AZ10606120 treatment had anti-depressant effects^11^ and reduced tumour growth^12^. The crystallization of zebrafish P2X4^13, 14^, Gulf Coast tick^15^ and human P2X3Rs has given structural insight to the ATP binding site and the orthosteric binding site of the P2X3 receptor antagonists TNP-ATP and A-317491^16^.

In this study, we have mapped the P2X7R AZ10606120 binding site and regions contributing to specificity by using chimeric receptors and a series of mutants. These studies coupled with *in silico* modelling have characterized residues important for AZ10606120 action, identified the location of a novel inter-subunit allosteric binding pocket at the apex of the receptor, and provide a model for the mode of allosteric inhibition of P2X7R by AZ10606120.

## Results

### A putative allosteric binding site in P2X7R

**Figure 1:**
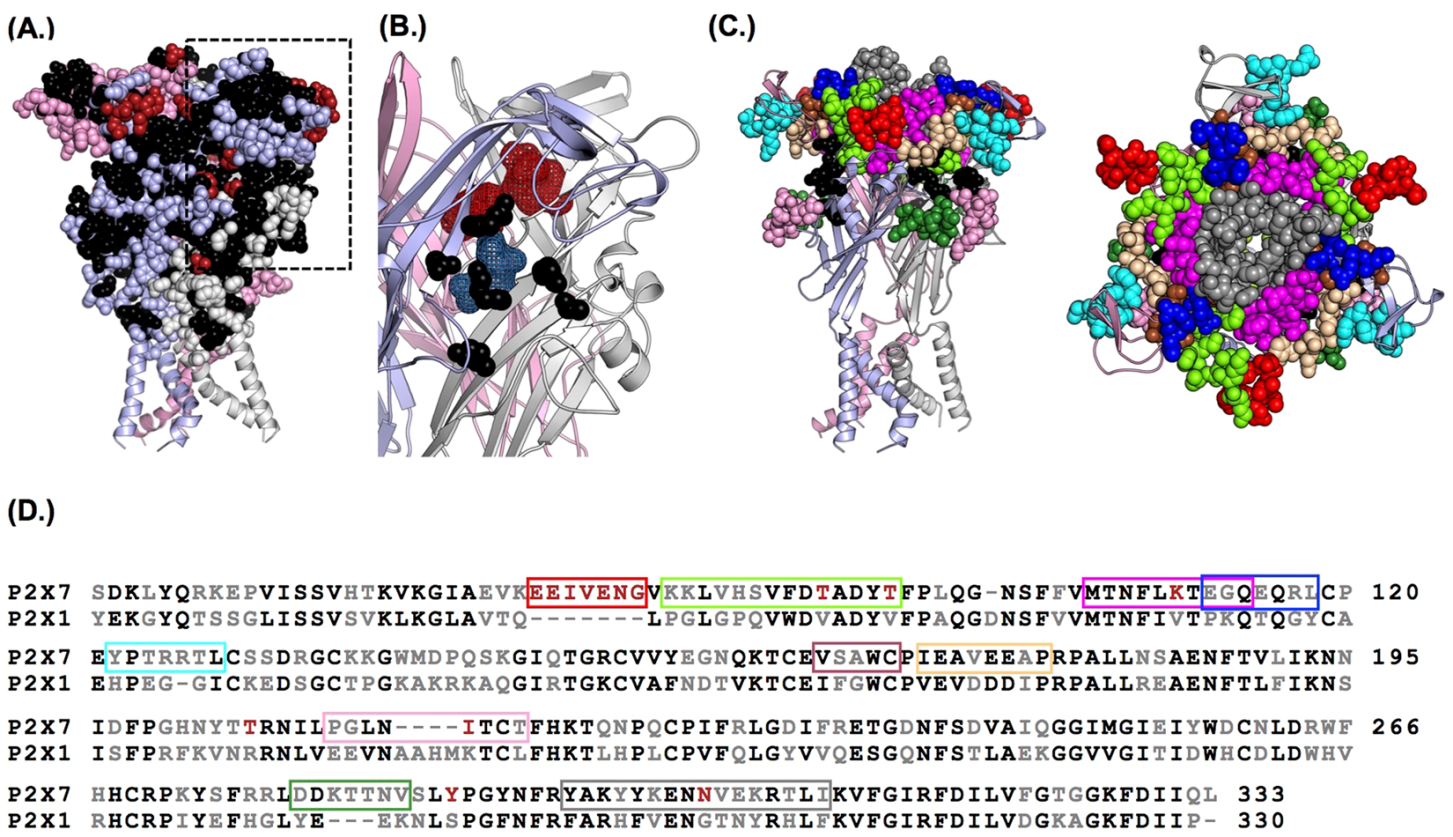
Location of chimeras and mutants to investigate potential orthosteric and allosteric binding sites for AZ10606120. (**A**) Homology model of the hP2X7R with the three subunits cartoon representations shown in grey, light purple and light pink. Conserved residues are shown as black spheres, residues unique to the P2X7R are shown as red spheres and variant residues between P2X7 and the other subunits are shown as spheres the colour of the subunit they are in. (**B**) FT site based predictions of orthosteric (teal) and allosteric (raspberry) binding pockets in the P2X7 receptor, conserved residues involved in ATP binding at one subunit interface are shown in black. Panel shows the region highlighted by dotted box in (**A**). (**C**) Homology model of the P2X7 receptor (from the side, left and from the top of the receptor, right) showing the region 73–79 (in red) and regions replaced by corresponding region of the P2X1 receptor for the chimeras (colour coding see (**D**)). Black spheres correspond to the residues that are conserved throughout P2X receptors that co-ordinate the binding of ATP. (**D**) Amino acid line-up of the extracellular ligand binding region of the human P2X7 and P2X1 receptors. Black residues are conserved in at least one mammalian orthologue of all P2X subunits, red is unique to the P2X7 receptor. Regions of mutation/chimera are shown by coloured boxes.

FTsite^17^ scans a protein structure with small probes to predict interaction/ligand binding sites. For the human (h) P2X7R this highlighted an orthosteric site and a putative allosteric site in the cavity at the subunit interface at the apex of the receptor (Fig. 1). Potential sites for antagonist action were further investigated by flexible ligand docking with RosettaLigand sampling the full extracellular region (Fig. S6). The majority of the 1000 ROSETTA poses with lowest energy are covered by the allosteric and orthosteric sites. More specifically, 60% of the docking poses were found in the putative allosteric site while 16% of the poses were found in an orthosteric site suggesting the allosteric site as most likely of AZ10606120 binding (the remaining 24% of the poses were distributed throughout the extracellular domain and not within any specific site). The combination of FTsite predictions and the first round of ligand docking results point towards AZ10606120 binding to an allosteric site, and provided the starting point for testing the molecular determinants of sensitivity and selectivity of AZ10606120 binding to P2X7R.

AZ10606120 shows >1,000 fold selectivity for the P2X7R over other P2XR subtypes^9, 10^. Sequence variation between the P2X7R and its paralogs must account for AZ10606120 specificity. While the P2X7R shares between 42% (with P2X6R) and 51% (with P2X4R) pairwise sequence identity with its paralogs in the extracellular ligand binding loop there are several features that are unique to the P2X7R and could contribute to antagonist selectivity; including a ≥7 amino acid insertion (residues 73–79 when compared to hP2X1) between two β-strands, a four amino acid deletion in the dorsal fin and several individual variant residues (Fig. S1). These differences are distributed over the surface of the receptor and many overlap with the potential orthosteric and allosteric binding sites (Fig. 1).

### Variations in the extracellular loop regions contribute to AZ10606120 sensitivity

**Figure 2:**
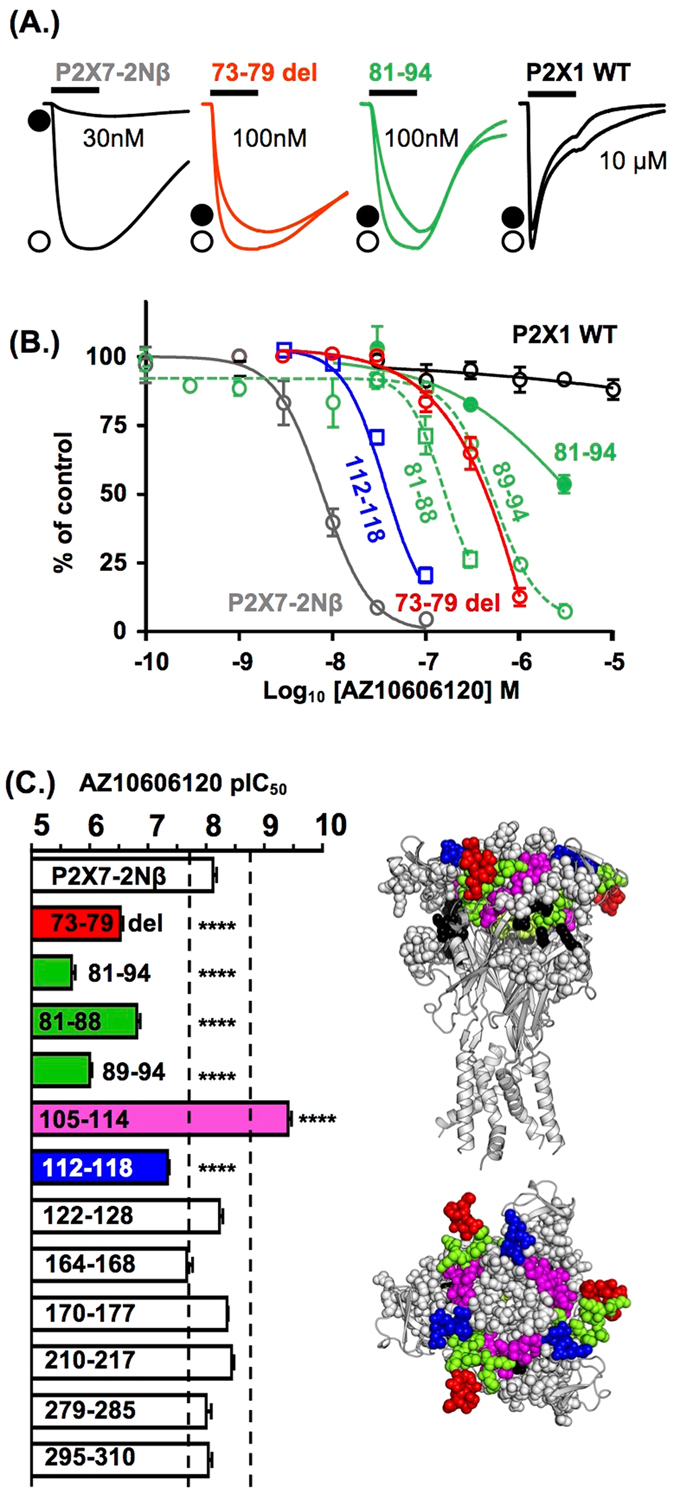
Identification of regions of the P2X7 receptor important for AZ10606120 sensitivity revealed using chimeras and the 73–79 deletion mutant. (**A**) Sample traces of currents evoked by an EC_90_ concentration of ATP (open circle) and in the presence of a range of concentrations of AZ10606120 (indicated on traces by filled circle) for P2X7–2Nβ, the P2X7 73–79 deletion mutant, the chimera replacing residues 81–94 of the P2X7 receptor with the corresponding sequence from the P2X1 receptor and the wild type P2X1 receptor. ATP was applied for 3 s (indicated by black bar). The currents for the different receptors/mutants are shown normalized to the peak response to allow comparisons between the effects of AZ10606120. The mean peak currents for these were 2.69 ± 0.19, 2.05 ± 0.23, 0.76 ± 0.13 and 7.90 ± 1.08 µA for P2X7-2Nβ, P2X7 73–79 del, 81–94 and P2X1 respectively. (**B**) Concentration dependent inhibition of ATP evoked responses by AZ10606120. (**C**) Summary of pIC_50_ values of AZ10606120 at mutant P2X7 receptors. A three-fold change from P2X7-2Nβ is indicated by the dotted line. Significance levels are shown only for those with >3 fold change in sensitivity. Right hand panels show the location of the mutants that had a >3 fold effect on AZ10606120 sensitivity, colour coding as in histogram for chimeras and conserved residues involved in ATP binding are shown in black. n = 3–7, ****p < 0.0001.

To determine the contribution of P2X7R specific residues, insertions and deletions around the predicted orthosteric and allosteric binding sites to AZ10606120 sensitivity we generated a deletion mutant and nine chimeric receptors (Fig. 1) that replaced regions of the P2X7R with the corresponding region of the hP2X1R that is insensitive to AZ10606120 (Fig. 2). Experimentally, P2X7Rs are distinct from other P2XRs in that responses to sequential applications of ATP show marked current facilitation both in rise time as well as peak amplitude and repeated ~60 s pulses of ATP are required to produce a stable fully facilitated response^18, 19^. We have previously shown that replacement of residues 16–26 of the intracellular amino terminus of the hP2X7 receptor with the corresponding region of the hP2X2 receptor (the hP2X7-2Nβ chimera) gives responses to ATP that quickly reach steady state (within 3 s) and are readily reproducible; features that expedite pharmacological characterization^19^. At the hP2X7-2Nβ receptor AZ10606120 inhibited responses evoked by an EC_90_ concentration of ATP (100 μM, in divalent cation free solution) in a concentration dependent manner with a pIC_50_ of 8.10 ± 0.05 (Fig. 2) and a Hill slope of 1.7 ± 0.24. This is equivalent to that at the hP2X7R^9^ and so we generated mutants and chimeras on the hP2X7-2Nβ background.

Deleting the unique P2X7R insertion (residues EEIVENG, 73–79 del) increased ATP sensitivity 2–3 fold (Table S1) and showed that the region does not make a major contribution to the low ATP potency of the hP2X7R. In all cases throughout this study any change in ATP sensitivity was corrected for in the antagonist testing by using an EC_90_ concentration of ATP^20^ and only mutants that resulted in ≥10 fold change in ATP sensitivity are commented on in the text (all values and significance are given in the Supplementary Tables). For these studies we have considered a >3 fold change in AZ10606120 sensitivity as potentially important (smaller, but statistically significant differences are shown in the Supplementary Tables). The 73–79 deletion reduced AZ10606120 sensitivity ~40 fold (pIC_50_ 6.53 ± 0.05, p < 0.0001) (Fig. 2). All the P2X7R/P2X1R chimeras were functional and the 279–285 chimera showed an ~10 fold increase in ATP sensitivity (p < 0.001) (Table S1). Swapping the regions 122–128, 164–168, 170–177, 210–217, 279–285 and 295–310 had <3 fold effect on AZ10606120 sensitivity (Fig. 2). AZ10606120 was ~20 fold more potent (p < 0.0001) at the 105–114 chimera. For the 112–118 chimera there was an ~6 fold decrease in AZ10606120 sensitivity (p < 0.0001). At the 81–94 chimera in the presence of 3 µM AZ10606120 the peak current to ATP was 53.6 ± 2.6% of the control response (estimated ~270 fold decrease in sensitivity based on a parallel shift in the antagonist inhibition curve). These findings highlight the importance of the 73–79 insertion and the region 81–94 in the antagonist action of AZ10606120 (Figs 2, S2, Table S1).

To determine the contribution of individual residues within the 73–79 insertion to antagonist action we generated individual cysteine point mutants. The level of inhibition for the 74–79 C mutants to 30 nM AZ10606120 (~70%) was the same as that for hP2X7–2Nβ (Table S2). The E73C mutant was non-functional so we generated the E73 single residue deletion (E73 del); this reduced AZ10606120 inhibition by ~20% (p < 0.05) (Table S2). These results demonstrate that individually residues 73–79 are unlikely to interact directly with AZ10606120 but suggest that the presence or absence of the entire loop has structural or dynamic effects on the receptor affecting AZ10606120 sensitivity.

**Figure 3:**
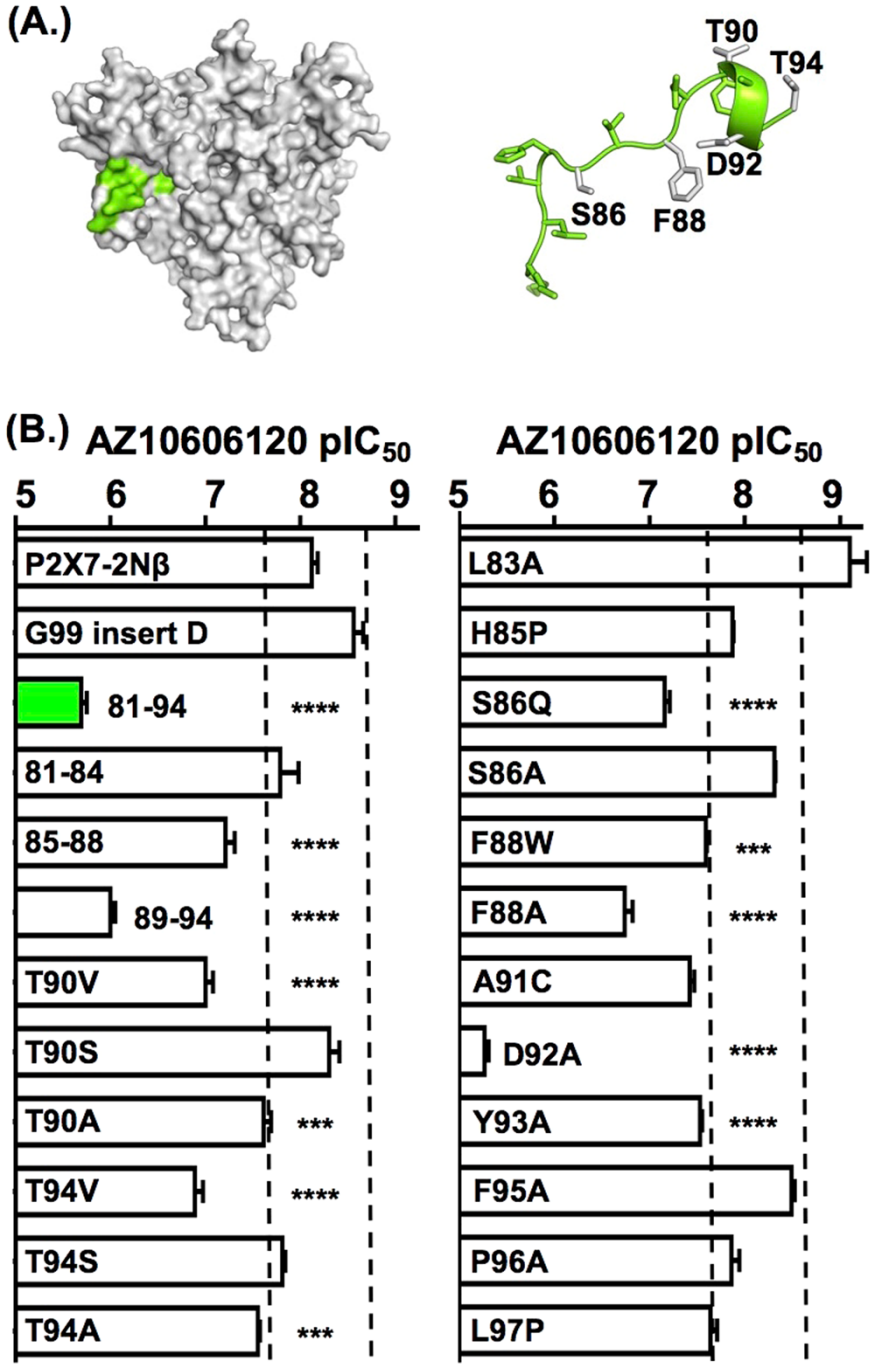
Contribution of variations in residues 81–94 to AZ10606120 sensitivity. (**A**) Homology model of the P2X7 receptor showing the location of the region 81–94 in one subunit, right hand panel shows cartoon and stick representation of this region (residues where mutation decreased AZ10606120 sensitivity are shown in grey). (**B**) Summary of pIC_50_ values of AZ10606120 at mutant P2X7 receptors. A three-fold change from P2X7-2Nβ is indicated by the dotted line. Significance levels are shown on the graph only for those with >3 fold change in sensitivity. n = 3–7, ***p < 0.001 ****p < 0.0001.

To investigate the contribution of the 81–94 region we generated a series of “sub-chimeras” (Figs 2, 3, S3, Table S1). For the 81–88 chimera there was an ~20 fold decrease in antagonist sensitivity (p < 0.0001). When this region was further divided the chimera 81–84 showed no change in AZ10606120 sensitivity and there was an ~10 fold decrease for the 85–88 chimera (p < 0.0001). The 89–94 chimera exhibited an ~130 fold decrease in AZ10606120 sensitivity (p < 0.0001, Fig. 3). This suggests that the two unique residues T90 and T94 in chimera 89–94 (conserved valines in all other P2XRs) make an important contribution to high affinity AZ10606120 antagonism. To test their individual contribution we generated T90V and T94V mutants; both of these resulted in an ~15 fold decrease (p < 0.0001) in AZ10606120 sensitivity (Fig. 3, Table S3). When the threonine was replaced by alanine there was an ~3 fold decrease in AZ10606120 sensitivity (p < 0.001). However the conservative substitution to serine, that maintained polarity with a small decrease in size of the side chain, did not change AZ10606120 sensitivity. These results suggest that polar amino acids at positions 90 and 94 are important for high affinity AZ10606120 binding.

### Modelling the effects of T90/T94 and the P2X7R unique insertion 73–79

Investigating the effect of variant regions identified the P2X7R unique residues T90 and T94, and the P2X7R unique insertion 73–79 as the main contributors to AZ10606120 binding. Mapping T90 and T94 onto the P2X7R homology model indicates that these residues are part of a short α-helix separating the orthosteric and putative allosteric site. To distinguish between potential allosteric and orthosteric binding modes nine point mutants of variant residues around the orthosteric site were tested (Figs S4, 5 and Table S4). These showed no effect on AZ10606120 sensitivity ruling out an orthosteric mode of action.

At a first glance one might expect that both, the P2X7R specific insertion 73–79, and the side-chains of T90 and T94 interact with AZ10606120 directly. However, T90 and T94 are ~25 Å distant from the P2X7R specific insertion 73–79, and the latter is a surface loop not part of the putative allosteric site. Considering this and the maximum distance of ~17 Å between two atoms within AZ10606120, direct interaction of AZ10606120 with both T90/T94 and the insertion 73–79 occurring at the same time seems unlikely. Also, T90 and T94 are adjacent to the allosteric binding site, but their side-chains are not directly pointing to the core of this pocket and in the ROSETTA docking process T90 and T94 sidechains do not tend to form hydrogen bonds with AZ10606120 (Fig. S6). These observations raise the question whether T90/T94 and/or the insertion 73–79 may affect AZ10606120 binding by modulating the structure and dynamics of the P2X7R rather than by direct interaction with the antagonist. To test this a series of 50 ns molecular dynamics simulations of P2X7R, P2X7R (T90V/T94V) and P2X7R (del73–79) (Fig. S7) was compared.

**Figure 4:**
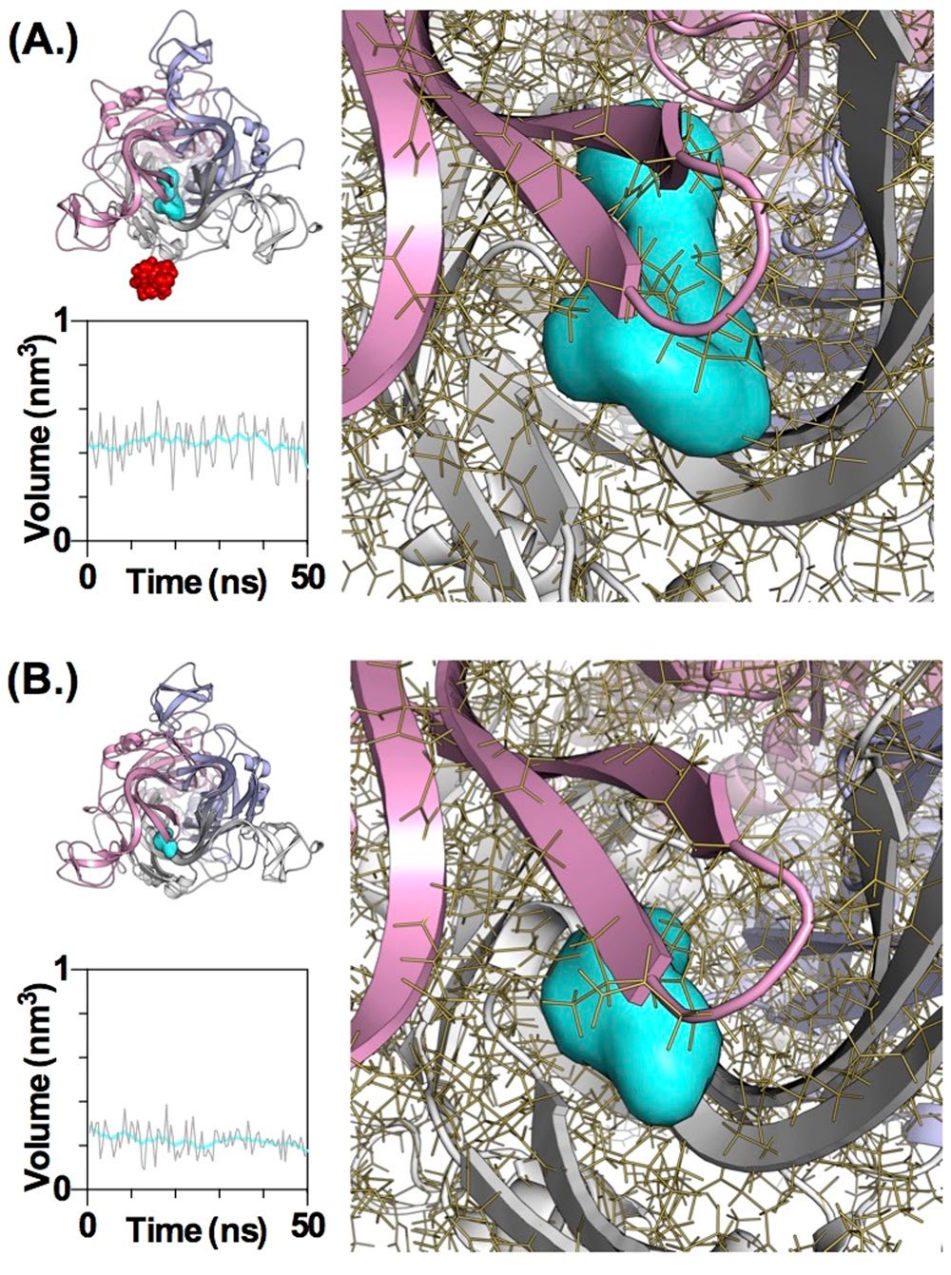
Molecular Dynamics Simulations of P2X7R and a P2X7R mutant with residues 73–79 deleted. (**A**) Variation of the size of the allosteric pocket in an MD simulation for the human P2X7R. A representative frame of the P2X7R MD simulation is shown in the top left panel (overview) and in the right panel (zoom). The P2X7R unique insertion region 73–79 is indicated as red spheres. The allosteric pocket as calculated in the mdpocket software is visualised as a cyan surface. In the bottom left panel the variation of the volume of the allosteric pocket during a 50 ns MD simulation derived from 100 frames is plotted as grey line, and with lowess fitting as the cyan line. (**B**) Variation of the size of the allosteric pocket in an MD simulation for the human P2X7R mutant without residues 73–79. Representation of data is as in (**A**).

In the wild-type P2X7R simulations T94 forms a persistent side-chain H-bond to the carbonyl group of T90 (78 ± 7% of frames) and in a fraction of the frames the T90 side-chain interacts with the hydroxyl-group of Y93 (10 ± 2% of frames). Both interactions cannot occur in the T90V/T94V mutant. As a consequence the short α-helix 90–93 is less persistent in the P2X7R (T90V/T94V) simulations and is present in only 34 ± 5% of the frames compared to 63 ± 10% in the P2X7R wild type simulations. Comparison with hP2X1 (47 ± 11%) suggests that the hydroxyl group side chains T90 and T94 in P2X7R effect differences in the dynamics of α-helix 90–93 deep in the allosteric pocket. Also the P2X7R unique insertion 73–79 is close to an anti-parallel β-sheet in proximity of the allosteric pocket (Fig. 4). Both observations strongly indicate that sequence variation unique to P2X7R may affect the receptor structure adjacent to the allosteric binding pocket. To determine more directly how these conformational changes might impact on the allosteric binding pocket we used MDpocket to extract pocket volumes from the MD simulations of P2X7R (del 73–79) and P2X7R (T90V/T94V), and compared them to P2X7R (Fig. 4, Table S5). Pocket sizes vary and the largest cavity is found in the wild type P2X7R with 410 ± 38 Å^3^. Interestingly the mean pocket volumes for both, P2X7R (del 73–79) and P2X7R (T90V/T94V) are significantly smaller with 301 ± 30 Å^3^ and 260 ± 33 Å^3^, (p < 0.05 and <0.01) respectively. While it is worth noting that these measurements are derived from the allosteric binding pocket in the closed state preceding AZ10606120 binding and not from the AZ10606120 bound state, the simulations provide evidence for a role of T90/T94 and the insertion 73–79 in shaping the allosteric pocket. These changes in size and shape may affect both, the access to allosteric pocket and the binding mode of AZ10606120, and hence the observed pattern of AZ10606120 antagonism.

### Mapping the allosteric binding pocket

**Figure 5:**
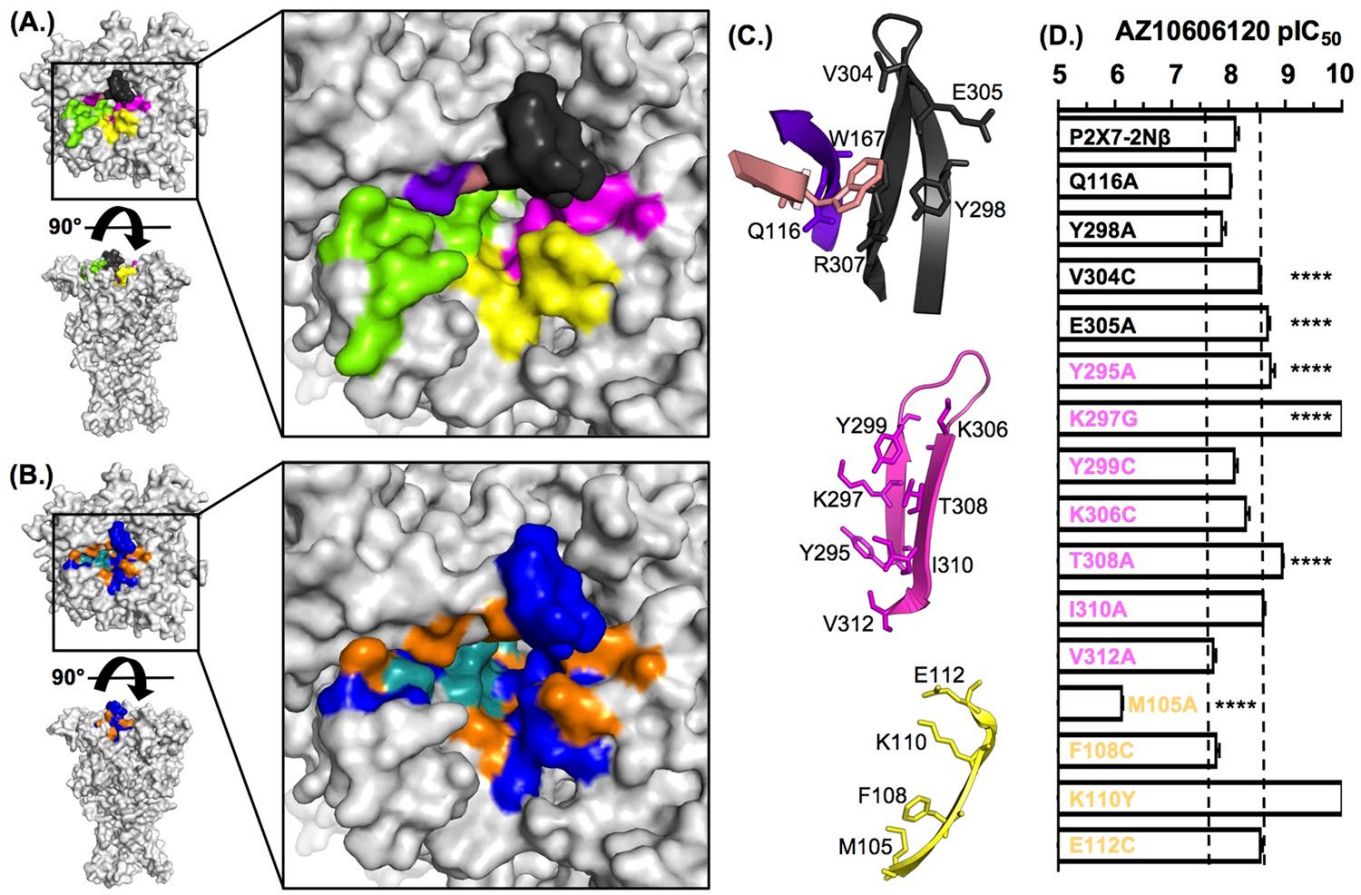
Contribution of variations around the putative allosteric binding pocket to AZ10606120 sensitivity. (**A**) The putative allosteric binding pocket is lined by residues from regions 81–94 (green), the loop 295–310 (magenta), strand 105–112 (yellow) and a series of other residues on the adjacent subunit (Q116 brown, W167 purple and residues on the other side of the loop 295–310 shown in black). (**B**) Individual point mutants with ≤3 fold change in AZ10606120 sensitivity are shown in orange, mutants that increased sensitivity are shown in blue and decrease AZ10606120 action in teal. (**C**) Cartoons showing the location of residues around the allosteric pocket. (**D**) Summary of pIC_50_ values of AZ10606120 at mutant P2X7 receptors. A three-fold change from P2X7-2Nβ is indicated by the dotted line. Significance levels are shown on the graph only for those with >3 fold change in sensitivity. n = 3–7, ****p < 0.0001.

The allosteric site forms from a series of structural elements. The 81–94 region contacts three β-strands comprising residues 105–112, as well as 295–299 and 306–312 that form a β-hairpin (Fig. 5). The remainder of the site is on the adjacent subunit, with a range of residues facing the pocket and thus potentially having a role in antagonist action. We therefore generated a series of point mutants of residues that line the allosteric binding pocket to determine the specific contribution of individual residues to antagonist sensitivity (Figs 3 and 5, Tables S3 and 6, Movie 1). Where there was variation between the P2X7 and P2X1Rs a mutation to the equivalent P2X1 residue was made. For residues conserved between P2X1&7Rs alanine or cysteine mutants were generated.

#### The 81–97 region

Mutation of the conserved leucine at position 83 (L83A) at the entrance to the allosteric binding pocket increased AZ10606120 sensitivity ~10 fold p < 0.0001) indicating that reducing the size of the residue may increase antagonist access. The S86Q mutation reduced AZ10606120 sensitivity ~10 fold (p < 0.0001), however the S86A mutant had no effect on AZ10606120 action. S86 is located at the entrance of the pocket, hence the increased size of the P2X1 residue substitution may interfere with AZ10606120 binding. In the F88 position there is an aromatic residue at all P2X receptors except P2X3. The F88W mutant reduced AZ10606120 sensitivity ~3 fold (p < 0.001). However, the F88A mutant decreased AZ10606120 sensitivity ~24 fold (p < 0.0001) highlighting the contribution of an aromatic residue at this position to the action of the antagonist. Mutations of D89 to either D89A or D89C were non-functional. The equivalent mutation in the rat P2X7R D89A was also non-functional (20). In the P2X4R the equivalent negatively charged residue forms a salt bridge with a conserved arginine (R307 P2X7R equivalent) that is important for receptor function (20). The A91C mutation showed a modest 5 fold decrease in AZ10606120 sensitivity (p < 0.0001) that may result from the more voluminous cysteine side chain (compared to the small methyl group of alanine), or increased polarity. Mutation of the conserved negatively charged D92 reduced AZ10606120 sensitivity by >700 fold. Y93 is conserved in all human P2X receptors with the exception of the P2X6R where it is a phenylalanine. The Y93A mutant had an ~4 fold decrease in AZ10606120 action (p < 0.0001), this residue faces away from the putative allosteric site and so the effects are likely to result from localised conformational disruption of the binding pocket. Mutation of the conserved proline at position 96 (P96A) had no effect on AZ10606120 sensitivity. L97 is unique to the P2X7R, mutations to alanine or cysteine were non-functional. However mutation to proline was tolerated and showed an ~3 fold decrease in AZ10606120 sensitivity. Finally, in this region we noticed that there was a unique deletion between residues 99 and 100 for the P2X7R. Insertion of an aspartic acid at this position (G99 insert D) increased AZ10606120 sensitivity ~3 fold (p < 0.01).

#### The 105–114 strand

The methionine at position 105 facing the allosteric pocket is conserved between P2X1 and P2X7Rs. AZ10606120 sensitivity was reduced ~100 fold at the M105A mutant (p < 0.0001). Removal of the positive charge at position 110 (K110Y) produced a large ~100 fold increase in sensitivity whilst removal of negative charge at position E112 (E112C) increased ~3 fold sensitivity to AZ10606120 (p < 0.001) (Fig. 5, Table S6). These results suggest that the charge and/or bulk of residues at positions 110 and 112 restrict access/reduce affinity of AZ10606120 to the allosteric pocket. These results further highlight the contribution of the strand 105–112 to the allosteric binding pocket.

#### The 295–310 β-hairpin

Removal of the conserved aromatic residue at position 295 (Y295A) increased ~4 fold sensitivity to AZ10606120 (p < 0.01). Substitution at position K297 with alanine or cysteine did not result in functional channels, however K297G was functional and increased AZ10606120 sensitivity >100 fold. There is a threonine at position 308 in all P2X receptors except P2X1, the T308A mutation showed an ~6 fold increase in AZ10606120 sensitivity. There was an ~3 fold change in sensitivity (increase and decrease respectively) for mutations at I310A and V312A (p < 0.001, p < 0.01 respectively).

#### Other residues lining the allosteric pocket

Residue 116 faces the allosteric pocket and is conserved in the human P2XRs. The Q116A mutant had no effect on AZ10606120 sensitivity (Fig. 5). Adjacent to Q116 in the structure is tryptophan 167 that is part of the WCP motif that is conserved throughout all P2XRs. Neither alanine, cysteine, phenylalanine nor serine mutants at position 167 were functional. At the rat P2X7R the equivalent alanine mutant was non-functional due to poor trafficking to the cell membrane (20). In close proximity to W167 is the conserved R307. However the R307 mutant was non-functional, this mutation abolishes the cation-π interaction between R307 and W167, consistent with mutation at the rP2X7R (20). Just “above” R307 are residues V304 and K305 that are part of the loop forming the hairpin between 295–299 and 306 to 312 (but these residues along with R307 point away from the allosteric pocket in that subunit). There was an ~3 fold increase in sensitivity for the V304C and E305A mutants (p < 0.0001). The final residue forming this lobe of the allosteric pocket, Y298 is conserved between the P2X7R and the P2X1 receptor. However, the alanine mutation at this position (Y298A) had a <2 fold effect on AZ10606120 sensitivity.

### Integrating mutagenesis and molecular modelling

The mutagenesis studies highlight the importance of both unique and conserved residues mediating the antagonist action of AZ10606120. Mapping the effect of the mutations onto the P2X7R homology model shows a cluster of residues with strong effects “lining” the allosteric pocket from the access region to deeper into the cavity (Fig. 5B). A second cluster of residues increasing AZ10606120 sensitivity is located around the opening of the allosteric pocket.

**Figure 6:**
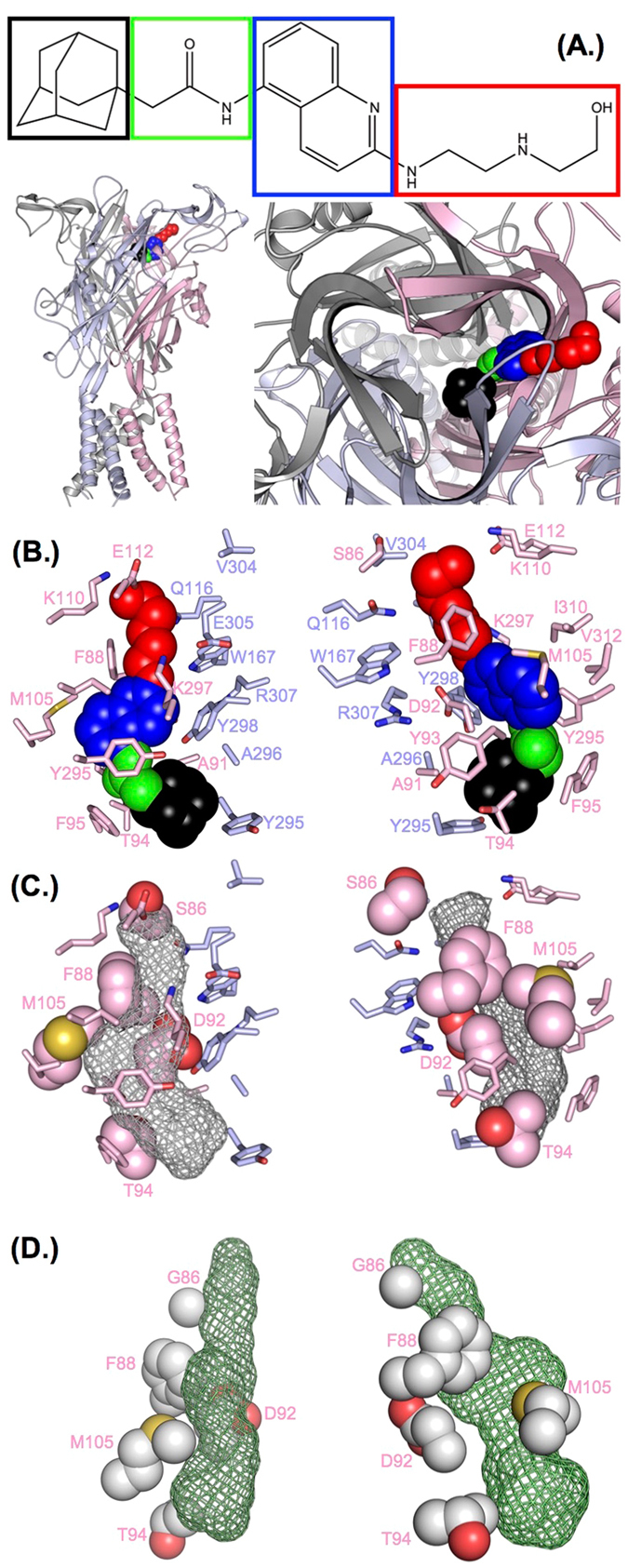
Comparison of docking of AZ10606120 into the allosteric pocket of the hP2X7R homology model and the panda P2X7R X-ray structure. (**A**) Top: Structure of AZ10606120. Regions referred to as adamantane, linker, aromatic and tail in the text are surrounded by black, green, blue and red boxes, respectively. This colour scheme is also used for AZ10606120 in the remaining panels of (**A**) and in (**B**). Bottom: The AZ10606120 docking pose in best agreement with experimental data is shown in an overview from a view parallel to the membrane (left), and in zoom perpendicular to the membrane (right). Light blue, light red and grey colours indicate different subunits. (**B**) P2X7R residues in proximity of AZ10606120 for the docking pose as in (**A**) are shown as sticks (side chain only) in two complementary views. Both views are derived by rotation from (**A**) bottom right. The colour scheme is as described in (**A**). (**C**) Residues for which mutation had the biggest effect on AZ10606120 inhibition are shown as spheres and labelled. The shape of AZ10606120 is indicated as grey mesh, both views are as in (**B**). (**D**) hP2X7R mutations that had the biggest effect on AZ10606120 inhibition were mapped on the P2X7R/AZ10606120 X-ray structure (PDB-entry: 5U1W (24)) and shown as spheres, AZ10606120 is represented as green mesh. Residue S86 in hP2X7R is equivalent to G86 in the panda P2X7R. Views in register as for (**B**) and (**C**).

A second round of targeted ROSETTA ligand docking focussed on the allosteric binding site was performed to enhance sampling, and analysed in the context of experimental data to provide a more detailed picture of AZ10606120/P2X7R interaction. The majority of the solutions fell into two clusters (39% and 37%) occupying the core of the putative allosteric site. While they overlapped in space they differed in the positioning of the adamantane moiety of AZ10606120 (top and bottom of the pocket). Both clusters were analysed to identify poses in which residues where mutations reduce AZ10606120 binding are in close proximity to the antagonist. In our favoured docking solution (Fig. 6, Movie 2) the unique residue F88 and the conserved residues D92 and M105 align the aromatic moiety of AZ10606120. The unique residue S86 at the gate of the pocket is in proximity of the AZ10606120 “tail”, while the aliphatic adamantane group sits deep in the binding pocket and is in proximity to T94. In summary, our combination of experiment and modelling provides a detailed structural analysis for the binding mode of the AZ10606120 inhibitor in a unique allosteric binding site.

## Discussion

In this study we used an integrated mutagenesis and *in silico* approach to map the binding site for the selective P2X7R antagonist AZ10606120. Initial molecular modelling suggested potential orthosteric and allosteric sites. Analysis of nine mutations of individual residues around the orthosteric site showed that AZ10606120 does not directly block ATP access to its binding site and ruled out an orthosteric mode of action. In contrast, chimeras and mutagenesis highlighted the interface between adjacent subunits at the apex of the receptor as important for AZ10606120 inhibition (Fig. 2C), and identified two features unique to the P2X7R affecting antagonist potency: a ≥7 amino acid P2X7R specific insertion (residues 73–79) and threonine residues at positions 90 and 94.

A second round of molecular docking focussed on the allosteric site allowed us to compare poses to the mutation data in more detail, and to divide mutants into two categories; those that are likely to interact directly with the antagonist and those that were predicted to have an indirect effect on the shape and dynamics of the allosteric pocket. For instance, mutations of F88, D92 and M105 significantly reduced AZ10606120 inhibition. These residues align the allosteric pocket and in the most probable docking poses interact directly with AZ10606120. However, these residues are conserved (or in case of F88 similar) between P2X receptor subtypes suggesting that while they contribute to binding, they do not encode specificity. For other residues aligning the allosteric pocket the introduction of smaller side-chains, e.g. in Y295A, K297G and T308A increased the affinity of AZ10606120 to P2X7R (Fig. 5), possibly by increasing the pocket size. A similar effect was seen for residues at the entrance of the pocket where an increase in side-chain size reduced AZ10606120 affinity (e.g. S86Q), possibly by limiting pocket access.

The side-chains of residues 90 and 94 are unique to P2X7Rs, but do not appear to have direct interaction with the antagonist raising the possibility that their mutation has structural effects on the conformation of the allosteric binding pocket. In MD simulations of P2X7R and the T90V/T94V mutant the T90/T94 side chains stabilise the short α-helix 90–93 and their mutation renders the dynamics of this region more hP2X1 like (see results). This dynamic effect coincides with a reduction of the average allosteric pocket volume, which in turn may affect binding of AZ10606120. Also the P2X7R unique insertion 73–79 contributes to antagonist sensitivity as its removal decreased AZ10606120 potency ~40 fold. Point mutations within this region had no effect on sensitivity to the antagonist suggesting that no individual residue contributes directly to AZ10606120 binding. This suggested that its deletion induces a conformational change in the receptor reducing antagonist sensitivity. Comparative MD simulations support this interpretation and show that this insert is likely to modulate the allosteric binding pocket by reducing the mean volume of the allosteric pocket.

Receptor selectivity often involves interactions of the antagonist with unique residues e.g. for the P2Y12 receptor for ADP the antagonist and antithrombotic agent clopidogrel inhibits bleeding by a biologically active metabolite binding to cysteine residues on the receptor^21^. However our study shows selectivity is unlikely to result from direct interactions of the antagonist with unique amino acids in the binding pocket as those that are predicted to directly interact with the antagonist and experimentally confirmed to affect AZ10606120 binding were conserved (or biophysically similar) in different P2X subtypes. Our results indicate that the unique features of the P2X7R that are associated with AZ10606120 do not produce selectivity directly by interacting with the antagonist but act indirectly, by modulation of the dynamic and structure of the pocket. As a consequence we propose that selectivity mostly comes from structural variations that affect access to/configuration of the allosteric pocket, rather than from residues within the allosteric pocket.

How does binding at the allosteric pocket lead to a decrease in ATP evoked current? Radioligand binding studies with AZ10606120 showed that ATP could inhibit binding of the antagonist^10^. However, the relationship was complex and it was suggested that ATP and AZ10606120 bind at separate but interacting sites on the P2X7R. This suggests that there may be conformational coupling between occupation of the orthosteric and access to the allosteric sites. These sites are connected by the short α-helix between T90 and T94. When AZ10606120 (~360 Å³ volume) is bound, the allosteric pocket cannot reduce to the volume found in the open state (171 ± 38 Å^3^). Therefore we propose that AZ10606120 plugs the allosteric pocket(s) and stabilizes a state of the receptor that restricts structural changes associated with ATP action and interferes with conformational coupling.

In summary we have mapped for the allosteric pocket of the P2X7R and provide a mechanism of action for antagonist inhibition of P2X7Rs. P2X7R selective antagonists have been suggested for targeting disease states where new therapies are needed, e.g. Alzheimer’s disease and pathologies where inflammation is a key feature^6^. Interestingly, recent virtual screening of the orthosteric site with a compound library identified new P2X7 antagonists^22^ and similar docking approaches and subsequent lead optimization have discovered a new therapeutic lead for opioid receptors^23^. Targeting the allosteric binding site of P2X7R reduces potential off-target effects and provides a template for rational drug design. When our paper was in the final stage of preparation a crystal structure of the P2X7R with AZ10606120 bound has been published^24^. Our prediction is consistent with, and complements the structural study. Both studies identified a new, largely hydrophobic allosteric binding site in P2X7R. Compared to our prediction from homology modelling, the crystal structure shows a different orientation of F95 at the bottom of the allosteric binding site and as a result the adamantane moiety of AZ10606120 sits slightly lower in the pocket. Our proposed mechanism for allosteric inhibition, in brief, the shrinking of the allosteric pocket required for activation can be blocked by binding of AZ10606120 is in full agreement with the X-ray structures and analysis by of Karasawa and Kawate, who also suggest that the allosteric pocket narrows upon ATP binding and that this conformational change is crucial for P2X7 channel activation. Our study has tested a comprehensive range of mutations of the residues lining the allosteric pocket and mapping these onto the structure is shown in Fig. 6e. Our results show that a combination of mutagenesis and modelling is able to make accurate predictions on the site of drug action. In addition we have identified that the unique P2X7R residues (the 73–79 loop and residues T90 and T94) are essential for high affinity antagonist binding and the structural changes they lead to (reduction in size of allosteric pocket) provide a mechanistic basis for the subtype selectivity of AZ10606120.

## Methods

### Generation of mutant P2X7 receptors

The hP2X1R was a gift from Dr Lin-Hua Jiang (University of Leeds, UK) and the hP2X1R cDNA has been described previously^25^. Chimeras were generated by megaprimer-mediated domain swapping^25^. In addition to chimeras, point mutations, deletions and insertions were made using the QuikChange (Stratagene) or Q5 (New England Bioscience) mutagenesis kits. The production of the correct mutations and absence of coding errors was verified by DNA sequencing (Automated ABI Sequencing Service, University of Leicester, UK).

### Expression in *Xenopus laevis* oocytes and electrophysiological recordings

P2XR constructs were transcribed to produce sense-strand cRNA (mMessage mMachine, Ambion, Austin, TX). Manually defoliculated stage V *Xenopus laevis* oocytes were injected with 50 nl (50 ng) of cRNA using an Inject + Matic microinjector (J.A. Gaby, InjectMatic, Geneva, Switzerland) and stored at 16 °C in ND96 buffer (in mM, NaCl 96, KCl 2, CaCl_2_ 1.8, MgCl_2_ 1, sodium pyruvate 5, HEPES 5 (pH 7.6) supplemented with 50 µg/ml gentamycin and 50 µg/ml tetracycline). For electrophysiological recordings (3–7 days post-injection) oocytes were bathed in divalent free ND96 buffer (in mM, NaCl 96, KCl 2, sodium pyruvate 5, HEPES 5 and 0.1 flufenamic acid, pH 7.6).

Two-electrode voltage clamp recordings were carried out using a GeneClamp 500B amplifier with a Digidata 1322 A analog to digital convertor and pCLAMP 8.2 acquisition software (Molecular Devices, Menolo Park, CA) at a holding potential of −60 mV. ATP (sodium salt, Sigma) was applied via a U-tube perfusion system for 3 s at 3–5 minute intervals to allow reproducible responses to be recorded. For determination of antagonist sensitivity AZ10606120 (Tocris) was bath perfused as well as co-applied with ATP through the U-tube. To standardize for any change in ATP sensitivity at mutant receptors antagonists were co-applied with an EC_90_ concentration of ATP.

In some cases chimeric or mutant P2X7Rs had reduced peak current amplitudes to ATP and/or a change in ATP sensitivity (indicated in tables). However even with a reduction robust responses could still be recorded and used to determine AZ10606120 sensitivity to an EC_90_ concentration of ATP. Therefore the underlying reason for any reductions in amplitude or changes in ATP sensitivity (less than three fold) are not commented on in the text and were not studied further.

### Homology modelling, ligand docking and molecular dynamics simulations

A series of 100 homology models for both, the closed and open forms of hP2X7 wild type covering the extracellular and TM domains was built in MODELLER v9.15^26^ and ranked according to their DOPE scores^27^. The closed and open forms of the zfP2X4 structure (PDB ID: 4DW0 and 4DW1) were used as templates. Models were also generated for hP2X7R (73–79 del), the hP2X7R (T90A, T94A) double mutant, and hP2X1R (all in the closed form). The best ranked models then served as the starting point for molecular dynamics (MD) simulations. The hP2X7R wild type model of the closed state was used to search in an unbiased way for potential antagonist binding sites with FTsite^17^.

MD simulations of hP2X7 models embedded in a lipid bilayer (DMPC, 332 molecules) were performed in AMBER14^28^ using the ff14SB, lipid14 and GAFF force fields. For MD simulations including the antagonist, RESP charges for AZ10606120 were derived using the HF/6-31 G* approach. Simulations were run in at least two replicates for 50 ns using random number seeds for the Langevin thermostat and the initial velocity. Trajectories were analysed using cpptraj^29^ and MDpocket^30^. For the wild type hP2X7R ten representative structures were extracted from the trajectories and used as starting conformations for ensemble ligand docking.

Ensemble ligand docking of AZ10606120 into the closed form of hP2X7R was carried out in RosettaLigand^31^. In a first round of docking a 30 Å sphere centred at the Cβ-atom of T94 was used to ensure the full extracellular domain was sampled. A second round of focussed docking used a 10 Å sphere around Cβ-atom of D92 to enhance sampling of the putative allosteric site. In both runs 10000 docked complexes of AZ10606120 with hP2X7 were generated. The top ten percent of the docked complexes (based on the lowest total ROSETTA energy) were further analysed by clustering using the amber cpptraj module. The structures with the lowest interface delta scores^32^ from the largest clusters were then visualised in PyMol for further analysis and comparison with the experimental data.

MDpocket^30^ was used to calculate pocket volumes for the allosteric pocket in the MD trajectories. The MD snapshots were aligned to a reference structure and extracted from the trajectory using the AMBER cpptraj module. MDpocket was run with the -L flag on the extracted snapshots. The output files contain a PyMol pse-file with conserved cavities during MD simulation, a frequency grid of 6.5 was used for filtering. Pockets corresponding to the putative allosteric binding site were then selected by visual inspection. MDpocket with -f flag and default settings was used to pass the pocket definition to the software for volume calculation. Pocket volumes were plotted with GraphPad Prism 6 (GraphPad software, San Diego, CA).

### Data analysis

Concentration response curves were normalized to the maximal response and fitted with the Hill equation (variable slope) with GraphPad Prism 6 (Graphpad software, San Diego, CA). ATP pEC_50_ is the −log_10_ of the concentration giving 50% of the maximal response (EC_50_ value). To quantify inhibition by AZ10606120 the effects of the antagonist are expressed as the % of the peak current amplitude to an EC_90_ concentration of ATP recorded before the application of antagonist (ATP gave reproducible responses to ATP in the absence of antagonist) data were fitted with GraphPad Prism 6. IC_50_ is the concentration of antagonist inhibiting the EC_90_ of ATP by 50%. pIC_50_ is −log_10_ of the IC_50_ value. Individual concentration response curves were generated for individual experiments and statistical analysis was carried out on the data generated. In the figures the inhibition curves are fitted to the mean normalized data. Any significant differences were calculated by one-way analysis of variance, followed by Bonferroni’s test (using GraphPad Prism 6). Data are shown as mean ± SEM. In all cases n ≥ 3 for all data points.

## Electronic supplementary material

Supplementary Tables and Figures

Movie 1

Movie 2
