# Supplementary material for: Unique residues in the ATP gated human P2X7 receptor define a novel allosteric binding pocket for the selective antagonist AZ10606120
Source: Sci Rep. 2017 Apr 7;7:725. doi: 10.1038/s41598-017-00732-5 (PMC5429621; doi:10.1038/s41598-017-00732-5)
Supplement: Supplementary file 1 — Supplementary Tables and Figures [file 41598_2017_732_MOESM1_ESM.pdf]

Unique residues in the ATP gated human P2X7 receptor define a novel allosteric binding pocket for the selective antagonist AZ10606120.

Short title: Mapping P2X7 receptor allosteric antagonist action.

Rebecca C. Allsopp[1], Sudad Dayl[1,2], Ralf Schmid[1,3] and Richard J. Evans[1*]

[1]Department of Molecular and Cell Biology, University of Leicester, Leicester, U.K. [2]Department of Chemistry, College of Science, University of Baghdad, Baghdad, Iraq. [3]Leicester Institute of Structural and Chemical Biology, University of Leicester, U.K.

	**Peak amplitude (μA)**	**ATP pEC_{50}**	**AZ10606120 pIC_{50}**	**pIC_{50} Fold shift**
**P2X7-2Nβ**	2.69 ± 0.19	4.39 ± 0.06	8.12 ± 0.06	-
**73-79 delete**	2.05 ± 0.23	4.79 ± 0.05 *	6.53 ± 0.05 ****	39.2
**81-94**	0.76 ± 0.13 ****	4.07 ± 0.05 *	5.69 ± 0.06 ****	269
**81-84**	1.31 ± 0.16 ****	4.89 ± 0.05 *	7.79 ± 0.19	2.1
**85-88**	2.32 ± .030	4.64 ± 0.07	7.21 ± 0.10 ****	8.1
**89-94**	1.86 ±0.16 **	4.36 ± 0.09	6.00 ± 0.04 ****	131.8
**105-114**	0.90 ± 0.10 ****	4.39 ± 0.06	9.41 ± 0.06 ****	← 19.5
**112-118**	1.74 ± 0.21 **	4.60 ± 0.07	7.34 ± 0.03 ****	6.0
**122-128**	1.13 ± 0.18 ****	4.34 ± 0.04	8.23 ± 0.06	1.3
**164-168**	2.69 ± 0.22	4.47 ± 0.11	7.67 ± 0.10 **	1.1
**170-177**	0.79 ± 0.16 ****	4.57 ± 0.01	8.36 ± 0.02	← 1.7
**210-217**	0.63 ± 0.17 ****	4.54 ± 0.04	8.44 ± 0.04 *	← 2.1
**279-285**	0.33 ± 0.01 ****	5.84 ± 0.20 ***	8.00 ± 0.09	1.3
**295-310**	0.78 ± 0.10 ****	3.99 ± 0.01 **	8.04 ± 0.06	1.2
**P2X1**	7.90 ± 1.08	6.06 ± 0.07	INSENSITIVE	-

Supplementary Table 1. Properties of mutant P2X7 receptors. The table shows the peak amplitude to a maximal concentration of ATP, the pEC_{50}, AZ10606120 pIC_{50} and fold shift in pIC_{50} for mutants relative to the P2X7-2Nβ receptor. Arrows for fold shift indicate where there was an increase in sensitivity to AZ10606120. All data are shown as mean ± SEM, n= 3-7. * $p<0.05$, ** $p<0.01$, *** $p<0.001$, **** $p<0.0001$.

	Peak amplitude (μA)	ATP pEC_{50}	% Inhibition with 30 nm AZ10606120
**2Nβ**	2.69 ± 0.19	4.39 ± 0.06	75.7 ± 3.28
**73-79**	2.05 ± 0.23	4.79 ± 0.05 *	6.67 ± 0.88 ****
**E73 del**	2.81 ± 0.09	4.78 ± 0.03 *	55.00 ± 2.00 *
**E74C**	1.20 ± 0.22	4.95 ± 0.12 ****	64.50 ± 3.85
**I75C**	0.61 ± 0.13 *	4.95 ± 0.05 ****	73.0 ± 4.62
**V76C**	0.98 ± 0.09 *	5.01 ± 0.03 ****	70.82 ± 3.82
**E77C**	0.28 ± 0.06 **	4.92 ± 0.05 ***	69.08 ± 1.53
**N78C**	0.36 ± 0.06 **	5.14 ± 0.03 ****	68.03 ± 3.34
**G79C**	2.4 ± 0.80	5.06 ± 0.10 ****	82.42 ± 4.73

Supplementary Table 2. Properties of mutant P2X7 receptors in the region 73-79. The table shows the peak amplitude to a maximal concentration of ATP, the pEC_{50}, and inhibition by 30 nM AZ10606120 of the response to an EC_{90} concentration of ATP. Arrows for fold shift indicate where there was an increase in sensitivity to AZ10606120. All data are shown as mean ± SEM, n= 3-7. * $p<0.05$.

	Peak amplitude (µA)	ATP pEC $_{50}$	AZ10606120 pIC_{50}	pIC $_{50}$ Fold shift
**2Nβ**	**2.69 ± 0.19**	**4.39 ± 0.06**	**8.12 ± 0.06**	
**L83A**	1.23 ± 0.19 ***	4.93 ± 0.04	9.10 ± 0.18 ****	← 9.5
**H85P**	1.53 ± 0.23	4.59 ± 0.19	7.87 ± 0.01	1.8
**S86A**	1.00 ± 0.24 *	4.70 ± 0.06	8.31 ± 0.01	1.6
**S86Q**	0.92 ± 0.18 ****	4.78 ± 0.11	7.16 ± 0.05 ****	9.1
**F88A**	0.83 ± 0.17 **	4.65 ± 0.04	6.74 ± 0.08 ****	24
**F88W**	1.07 ± 0.18 ***	4.67 ± 0.07	7.59 ± 0.01 ***	3.4
**T90A**	1.78 ± 0.27	4.62 ± 0.07	7.61 ± 0.08 ***	3.2
**T90S**	1.58 ± 0.21	4.75 ± 0.05	8.30 ± 0.11	1.5
**T90V**	3.25 ±0.40	4.47 ± 0.04	7.0 ± 0.08 ****	13.2
**A91C**	0.12 ± 0.02 ****	4.25 ± 0.10 *	7.42 ± 0.05 ****	5.0
**D92A**	0.70 ± 0.16 ****	4.73 ± 0.13	5.27 ± 0.04 ****	708
**D92G**	0.56 ± 0.20 **	4.58 ± 0.09	< 3	INSENSITIVE
**D92R**	0.50 ± 0.06 ****	5.33 ± 0.13	< 3	INSENSITIVE
**Y93A**	0.49 ± 0.05 ****	3.97 ± 0.04	7.53 ± 0.02 ****	3.9
**T94A**	1.58 ± 0.21	4.56 ± 0.03	7.55 ± 0.02 ***	3.7
**T94S**	1.53 ± 0.34	4.68 ± 0.01	7.81 ± 0.03	2.0
**T94V**	3.18 ± 0.41	4.66 ± 0.01	6.89 ± 0.08 ****	17.0
**F95A**	2.58 ± 0.26	3.91 ± 0.06 ****	8.49 ± 0.04 *	← 2.3
**P96A**	3.18 ± 0.42	4.50 ± 0.05	7.86 ± 0.08	1.8
**L97P**	2.13 ± 0.31	5.84 ± 0.10 ****	7.64 ± 0.07 ***	3.0
**Insert**	2.26 ± 0.40	4.21 ± 0.03 *	8.56 ± 0.10 **	← 2.8

Supplementary Table 3. Properties of point mutant P2X7 receptors in the region 83-97. The table shows the peak amplitude to a maximal concentration of ATP, the pEC_{50}, AZ10606120 pIC_{50} and fold shift in pIC_{50} for mutants relative to the P2X7-2Nβ receptor. Residue V87 is conserved in hP2X1&7Rs but faces away from the putative allosteric binding pocket in the homology model and so was not investigated. At position 97 the leucine residue is unique to the P2X7R, mutations to alanine or cysteine were non-functional. However proline substitution was tolerated (L97P) and resulted in a small ~ 3 fold decrease in AZ10606120 sensitivity ($p<0.001$). Arrows for fold shift indicate where there was an increase in sensitivity to AZ10606120. All data are shown as mean ± SEM, n= 3-7. * $p<0.05$, ** $p<0.01$, *** $p<0.001$, **** $p<0.0001$.

	Peak amplitude (µA)	ATP pEC $_{50}$	AZ10606120 pIC$_{50}$	pIC $_{50}$ Fold shift
**2Nβ**	2.69 ± 0.19	4.39 ± 0.06	8.12 ± 0.06	-
**H62S**	1.15 ± 0.19 ****	4.579 ± 0.115	8.58 ± 0.10 ***	← 2.9
**F95A**	2.58 ± 0.26	3.89 ± 0.08 **	8.50 ± 0.04 ***	← 2.4
**Q143K**	1.66 ± 0.43	4.63 ± 0.19	8.44 ± 0.035 **	← 2.1
**S144A**	1.87 ± 0.30	5.07 ± 0.04	7.93 ± 0.02	1.6
**Y288S**	1.40 ± 0.14 ***	4.05 ± 0.17 *	8.37 ± 0.02 *	← 1.8
**Y291A**	0.98 ± 0.97 *	4.84 ± 0.04	7.92 ± 0.12	1.6
**Y291F**	2.30 ± 0.32	4.26 ± 0.78	8.05 ± 0.04	1.2
**Y295A**	0.17 ± 0.04****	4.16 ± 0.77	8.74 ± 0.08 ****	← 4.2
**Y295F**	2.87 ± 0.31	4.28 ± 0.18	8.47 ± 0.04 **	← 2.2
**P2X1**	7.90 ± 1.08	6.057 ± 0.072	INSENSITIVE	-

Supplementary Table 4. Properties of mutant P2X7 receptors around the orthosteric binding pocket. The table shows the peak amplitude to a maximal concentration of ATP, the pEC$_{50}$, AZ10606120 pIC$_{50}$ and fold shift in pIC$_{50}$ for mutants relative to the P2X7-2Nβ receptor. Arrows for fold shift indicate where there was an increase in sensitivity to AZ10606120. All data are shown as mean ± SEM, n= 3-7. * p<0.05, ** p<0.01, *** p<0.001, **** p<0.0001.

P2X7R closed (wild type)	P2X7R closed (del 73-79)	P2X7R closed (T90V/T94V)	P2X1R closed (wild type)	P2X7R open (wild type)
535	464	313	213	108
530	396	230	258	135
302	305	198	114	35
541	201	351	334	69
245	224	244	58	87
449	373	219	474	183
333	277	457	447	341
448	244	196	85	340
306	223	130	321	241
Mean ± SE 410 ± 38	Mean ± SE 301 ± 30*	Mean ± SE 260 ± 33**	Mean ± SE 256 ± 51*	Mean ± SE 171 ± 38**

Supplementary Table 5. Volumes of allosteric pocket. Volumes are reported in $Å^3$. Measurements were taken from three independent molecular dynamics runs for each receptor state using MDpocket. Due to the trimeric receptor each run results three volume measurements. P-values are reported relative to the P2X7R closed, wild-type data (* $p<0.05$, ** $p<0.01$).

	Peak amplitude (μA)	ATP pEC_{50}	AZ10606120 pIC_{50}	pIC_{50} Fold shift
**2Nβ**	2.69 ± 0.19	4.39 ± 0.06	8.12 ± 0.06	-
**M105A**	1.88 ± 0.39 ****	3.84 ± 0.05 ****	6.12 ± 0.02 ****	100
**F108C**	0.18 ± 0.021 ****	4.71 ± 0.05	7.78 ± 0.06 ***	2.2
**K110Y**	0.59 ± 0.094 ****	4.23 ± 0.06	> 10	
**E112C**	0.28 ±0.038 ****	4.50 ± 0.16	8.56 ± 0.04 ***	← 2.8
**Y295A**	0.17 ± 0.04 ****	4.16 ± 0.77 ****	8.74 ± 0.08 ****	← 4.2
**Y295F**	2.87 ± 0.31	4.28 ± 0.18	8.47 ± 0.04 **	← 2.2
**K297G**	0.42 ± 0.06	4.08 ± 0.02	> 10	
**Y298A**	0.20 ± 0.05 ****	3.87 ± 0.47 ****	7.88 ± 0.07 *	1.7
**Y299C**	0.38 ± 0.04 ****	3.83 ± 0.40 ****	8.10 ± 0.06	1.03
**K306C**	0.89 ± 0.14 ****	4.66 ± 0.89	8.30 ± 0.07	← 1.5
**T308**	0.20 ± 0.05	4.99 ± 0.11	8.95 ± 0.02 ****	← 6.8
**I310A**	0.78 ± 0.14 ***	4.43 ± 0.01	8.61 ± 0.04 ****	← 3.0
**V312A**	1.15 ± 0.20 *	4.40 ± 0.03	7.73 ± 0.05 **	2.5
**Q116A**	0.33 ± 0.07 ****	3.90 ± 0.03 ***	8.03 ± 0.02	1.2
**R117C**	0.27 ± 0.09 ****	4.33 ± 0.02	8.45 ± 0.04 **	← 2.1
**A166G**	0.81 ± 0.15 ****	5.21 ± 0.05 *	7.65 ± 0.05 ***	← 2.9
**Y298A**	0.20 ± 0.05 ****	3.87 ± 0.47	7.88 ± 0.07 *	1.7
**V304C**	0.62 ± 0.07 ****	4.54 ± 0.47	8.54 ± 0.03 ****	← 2.6
**E305A**	1.04 ± 0.16 ****	4.58 ± 0.14	8.69 ± 0.04 ****	← 3.7

Supplementary Table 6. Properties of mutant P2X7 receptors around the allosteric binding pocket. The table shows the peak amplitude to a maximal concentration of ATP, the pEC_{50}, AZ10606120 pIC_{50} and fold shift in pIC_{50} for mutants relative to the P2X7-2Nβ receptor. Arrows for fold shift indicate where there was an increase in sensitivity to AZ10606120. All data are shown as mean ± SEM, n=3-7. * $p<0.05$, ** $p<0.01$, *** $p<0.001$, **** $p<0.0001$.

```
P2X1   -YEKGYQTSSGLI-SSVSVKLKGLAVTQ-------LPGLGPQVWDVADYV  91
hP2X7  -SDKLYQRKEPVI-SSVHTKVKGIAEVKEEIVENGVKKLVHSVFDTADYT  94
rP2X7  -SDKLYQRKEPLI-SSVHTKVKGVAEVTENVTEGGVTKLVHGIFDTADYT  94
P2X2   ---KSYQDSETGPESSIITKVKGITTSE------------HKVWDVEEYV  87
P2X3   LHEKAYQVRDTAIESSVVTKVKGSGLYA------------NRVMDVSDYV  81
P2X4   ---KGYQETDSVV-SSVTTKVKGVAVTNTS-------KLGFRIWDVADYV  90
zP2X4  ---KGYQDTDTVL-SSVTTKVKGIALTNTS-------ELGERIWDVADYI  93
P2X5   --KKGYQDVDTSLQSAVITKVKGVAFTNT-------SDLGQRIWDVADYV  92
P2X6   --KKGYQERDLEPQFSIITKLKGVSVTQI-------KELGNRLWDVADFV  91

P2X1   FPAQGDNSFVVMTNFIVTPKQTQGYCAEHPE-G-GICKEDSGCTPGKAKR 139
hP2X7  FPLQG-NSFFVMTNFLKTEGQEQRLCPEYPT-RRTLCSSDRGCKKGWMDP 142
RP2X7  LPLQG-NSFFVMTNYLKSEGQEQKLCPEYPS-RGKQCHSDQGCIKGWMDP 142
P2X2   KPPEGGSVFSIITRVEATHSQTQGTCPESIRVHNATCLSDADCVAGELDM 137
P2X3   TPPQGTSVFVIITKMIVTENQMQGFCPESE--EKYRCVSDSQC--GPEPL 127
P2X4   IPAQEENSLFVMTNVILTMNQTQGLCPEIPD-ATTVCKSDASCTAGSAGT 139
zP2X4  IPPQEDGSFFVLTNMIITTNQTQSKCAENPT-PASTCTSHRDCKRGFNDA 142
P2X5   IPAQGENVFFVVTNLIVTPNQRQNVCAENEGIPDGACSKDSDCHAGEAVT 142
P2X6   KPPQGENVFFLVTNFLVTPAQVQGRCPEHPSVPLANCWVDEDCPEGEGGT 141

P2X1   KAQGIRTGKCVAF-NDTVKTCEIFGWCPVEVDDDIPRPALLREAENFTLF 188
hP2X7  QSKGIQTGRCVVH-EGNQKTCEVSAWCPIEAVEEAPRPALLNSAENFTVL 191
rP2X7  QSKGIQTGRCIPY-DQKRKTCEIFAWCPAEEGKEAPRPALLRSAENFTVL 191
P2X2   LGNGLRTGRCVPYYQGPSKTCEVFGWCPVE-DGASDSQFLGTMAPNFTIL 186
P2X3   PGGGILTGRCVNY-SSVLRTCEIQGWCPTEVDT-VETPIM-MEAENFTIF 174
P2X4   HSNGVSTGRCVAF-NGSVKTCEVAAWCPVEDDTHVPQPAFLKAAENFTLL 188
zP2X4  RGDGVRTGRCVSY-SASVKTCEVLSWCPLEKIVDPPNPPLLADAENFTVL 191
P2X5   AGNGVKTGRCLRRGNLARGTCEIFAWCPLETSS-RPEEPFLKEAEDFTIF 191
P2X6   HSHGVKTGQCVVFNGTHR-TCEIWSWCPVESGV-VPSRPLLAQAQNFTLF 189

P2X1   IKNSISFPRFKVN-RRNLVEEVNAAHMKTCLFHKTLHPLCPVFQLGYVVQ 237
hP2X7  IKNNIDFPGHNYTTR-NILPGLNI----TCTFHKTQNPQCPIFRLGDIFR 236
rP2X7  IKNNIDFPGHNYTTR-NILPGMNI----SCTFHKTWNPQCPIFRLGDIFQ 236
P2X2   IKNSIHYPKFHFS-KGNIADR-TDGYLKRCTFHEASDLYCPIFKLGFIVE 234
P2X3   IKNSIRFPLFNFE-KGNLLPNLTARDMKTCRFHPDKDPFCPILRVGDVVK 223
P2X4   VKNNIWYPKFNFS-KRNILPNITTTYLKSCIYDAKTDPFCPIFRLGKIVE 237
zP2X4  IKNNIRYPKFNFN-KRNILPNINSSYLTHCVFSRKTDPDCPIFRLGDIVG 240
P2X5   IKNHIRFPKFNFS-KNNVMDVKDRSFLKSCHFGPK-NHYCPIFRLGSIVR 239
P2X6   IKNTVTFSKFNFS-KSNALETWDPTYFKHCRYEPQFSPYCPVFRIGDLVA 238

P2X1   ESGQNFSTLAEKGGVVGITIDWHCDLDWHVRHCRPIYEFHGLYE---EKN 284
hP2X7  ETGDNFSDVAIQGGIMGIEIYWDCNLDRWFHHCHPKYSFRRLDDKTTNVS 286
rP2X7  EIGENFTDVAVQGGIMGIEIYWDCNLDSWSHRCQPKYSFRRLDDKYTNES 286
P2X2   KAGENFTELAHKGGVIGVIINWDCDLDLPASECNPKYSFRRLDPKHV--P 283
P2X3   FAGQDFAKLARTGGVLGIKIGWVCDLDKAWDQCIPKYSFTRLDSVSEKSS 273
P2X4   NAGHSFQDMAVEGGIMGIQVNWDCNLDRAASLCLPRYSFRRLDTRDVEHN 287
zP2X4  EAEEDFQIMAVHGGVMGVQIRWDCDLDMPQSWCVPRYTFRRLDNKDPDNN 290
P2X5   WAGSDFQDIALRGGVIGINIEWNCDLDKAASECHPHYSFSRLDNKLSKS- 288
P2X6   KAGGTFEDLALLGGSVGIRVHWDCDLDTGDSGCWPHYSF-QLQEK----- 282

P2X1   LSPGFNFRFARHFVE-NGTNYRHLFKVFGIRFDILVDGKAGKFDIIP- 330
hP2X7  LYPGYNFRYAKYYKE-NNVEKRTLIKVFGIRFDILVFGTGGKFDIIQL 333
rP2X7  LFPGYNFRYAKYYKE-NGMEKRTLIKAFGVRFDILVFGTGGKFDIIQL 333
P2X2   ASSGYNFRFAKYYKI-NGTTTRTLIKAYGIRIDVIVHGQAGKFS---- 326
P2X3   VSPGYNFRFAKYYKMENGSEYRTLLKAFGIRFDVLVYGNAGKFN---- 317
P2X4   VSPGYNFRFAKYYRDLAGNEQRTLIKAYGIRFDIIVFGKAGKFDIIPT 335
zP2X4  VAPGYNFRFAKYYKNSDGTETRTLIKGYGIRFDVMVFGQAGKFNIIPT 338
P2X5   VSSGYNFRFARYYRDAAGVEFRTLMKAYGIRFDVMVNGK-GAFF---- 331
P2X6   ---SYNFRTATHWWEQPGVEARTLLKLYGIRFDILVTGQAGKFG---- 323
```

Supplementary Figure 1. Sequence alignment of the extracellular loop of human P2X receptors. Alignment of human and rat (rP2X7) receptors. Colour coding; conserved in all (red) conserved for P2X1 and P2X7Rs (green), unique to P2X7 (orange).

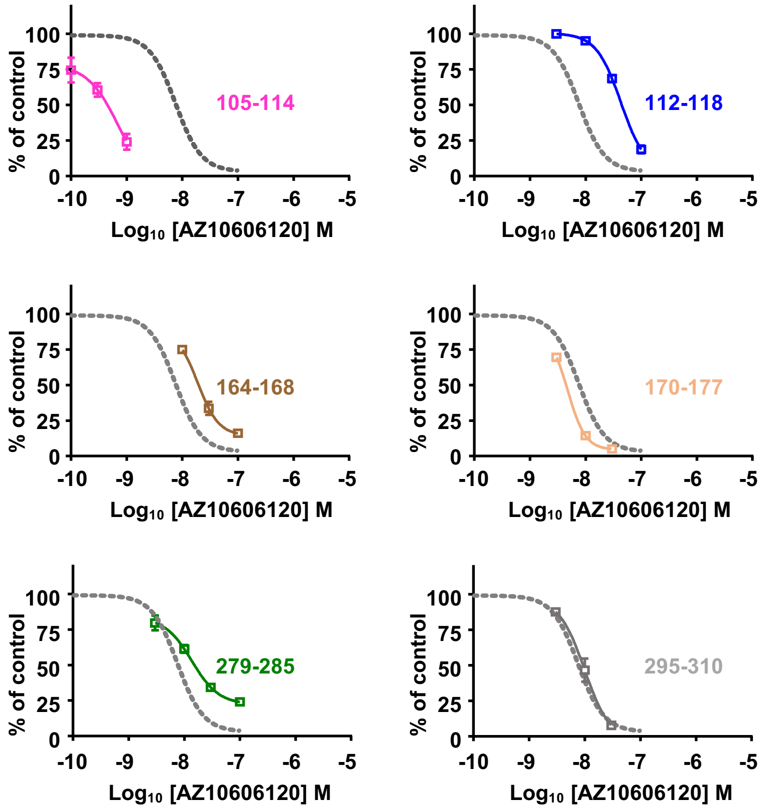

Supplementary Figure 2. Concentration dependence of inhibition of ATP responses by AZ10606120 at chimeric receptors. Histograms show the inhibition (mean ± SEM) of response to an EC_{90} concentration of ATP by AZ10606120. Grey dotted line corresponds to the inhibition at the P2X7-2Nβ receptor. Colour coding of traces is as shown in Figure 1B. n= 3-7.

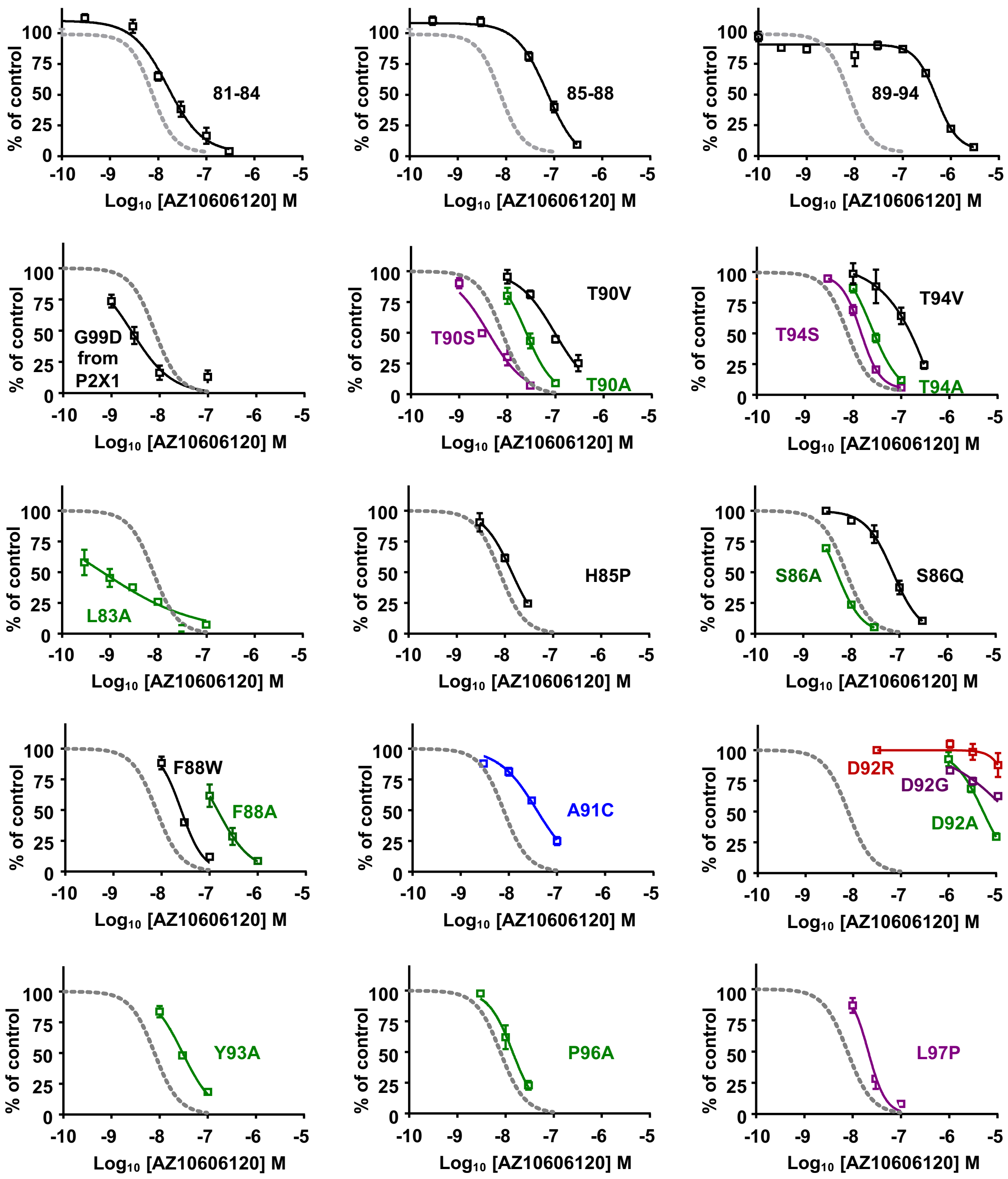

Supplementary Figure 3. Concentration dependence of inhibition of ATP responses by AZ10606120 at sub-chimeras and point mutants in the region 83-97. Histograms show the inhibition (mean ± SEM) of response to an EC_{90} concentration of ATP by AZ10606120. Grey dotted line corresponds to the inhibition at the P2X7-2Nβ receptor. Black traces indicate where the amino acid from P2X7R is replaced by that from P2X1R. Green corresponds to an alanine mutation. Purple corresponds to other point mutations. n=3-7.

(A.)

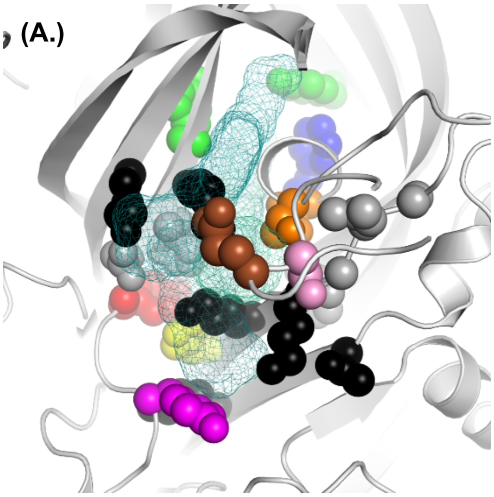

(B.)

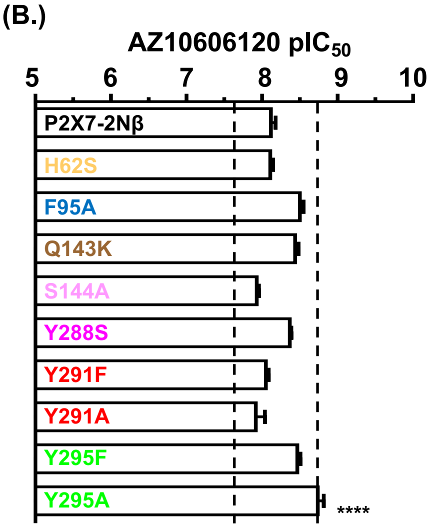

Supplementary Figure 4. Individual variations around the orthosteric binding site do not account for AZ10606120 sensitivity. The orthosteric site shows a high degree of conservation throughout mammalian P2XR subtypes with only a few variant residues identified in the predicted allosteric binding poses for AZ10606120; Q143 (that is unique to P2X7Rs), S144 (that is also found in hP2X4&6), H62 (unique to P2X7), Y288 (aromatic residue unique to P2X7), and tyrosines 291 and 295 (conserved aromatic throughout mammalian P2Xs). At the back of the orthosteric pocket is residue F95 that has been shown to contribute to the species dependent selectivity of the antagonist AZ11645373 (Michel et al, 2008, 2009). This residue is also a phenylalanine at the hP2X1R. We swapped the hP2X7R residue for the corresponding one from the hP2X1R, and for conserved aromatic groups also made alanine mutants. For all the mutants there was no, or <3 fold change in AZ10606120 sensitivity, with the exception of the Y295A mutant that increased sensitivity ~4 fold (Supplementary Table 4). These results show that individual variations in the orthosteric binding pocket are unlikely to account for AZ10606120 selectivity at the P2X7R and indicate that the AZ10606120 mode of action is not orthosteric. (A.) Homology model of the P2X7 receptor showing the orthosteric binding pocket. Residues throughout the P2X receptor family that co-ordinate the binding of ATP are shown as grey spheres. P2X7 variant residues around the orthosteric pocket are shown in colour. Docking of AZ10606120 is shown in grey mesh. Conserved residues involved in ATP binding are in black and other residues that are conserved around the pocket are shown in grey. (B.) Summary of pIC$_{50}$ values of AZ10606120 at mutant P2X7 receptors. A three fold change from P2X7-2Nβ is indicated by the dotted line. Significance levels are shown on the graph only for those with >3 fold change in sensitivity. n= 3-7, **** $p<0.0001$.

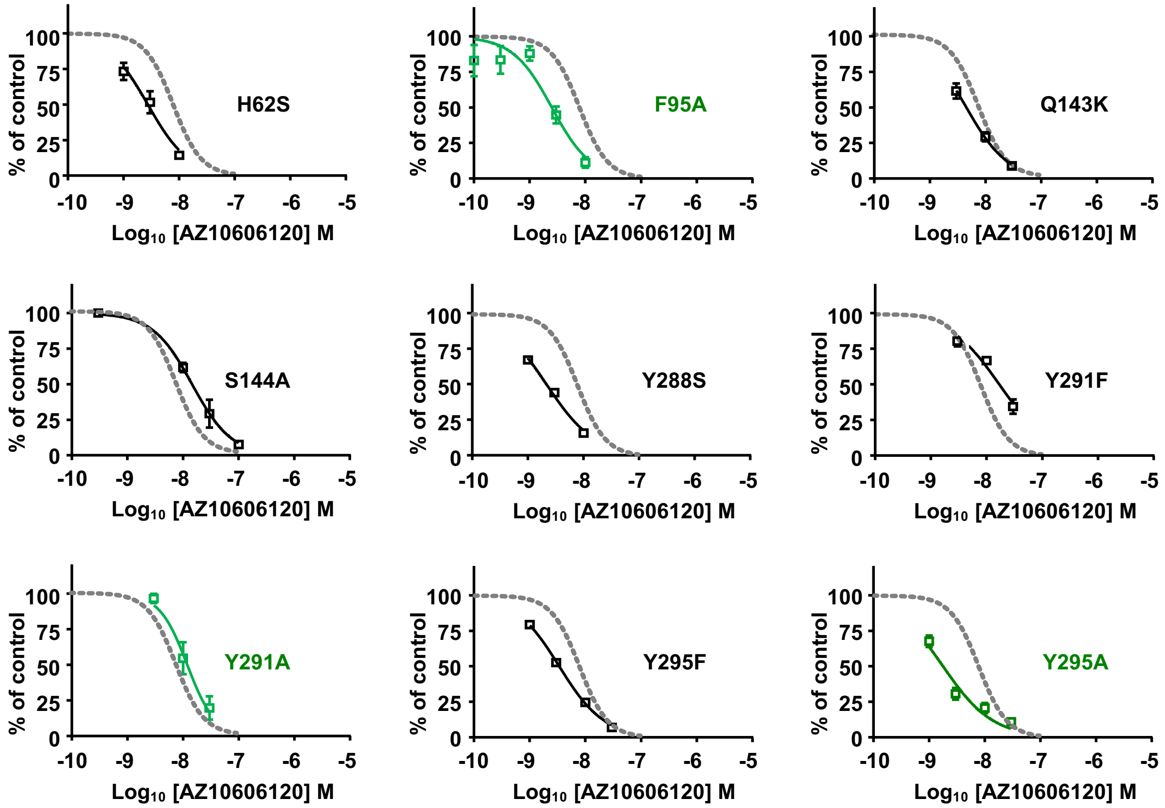

Supplementary Figure 5. Concentration dependence of inhibition of ATP responses by AZ10606120 at point mutants around the orthosteric binding site. Histograms show the inhibition (mean ± SEM) of response to an EC_{90} concentration of ATP by AZ10606120. Grey dotted line corresponds to the inhibition at the P2X7-2Nβ receptor. Black traces indicate where the amino acid from P2X7R is replaced by that from P2X1R. Green corresponds to an alanine mutation. n= 3-7.

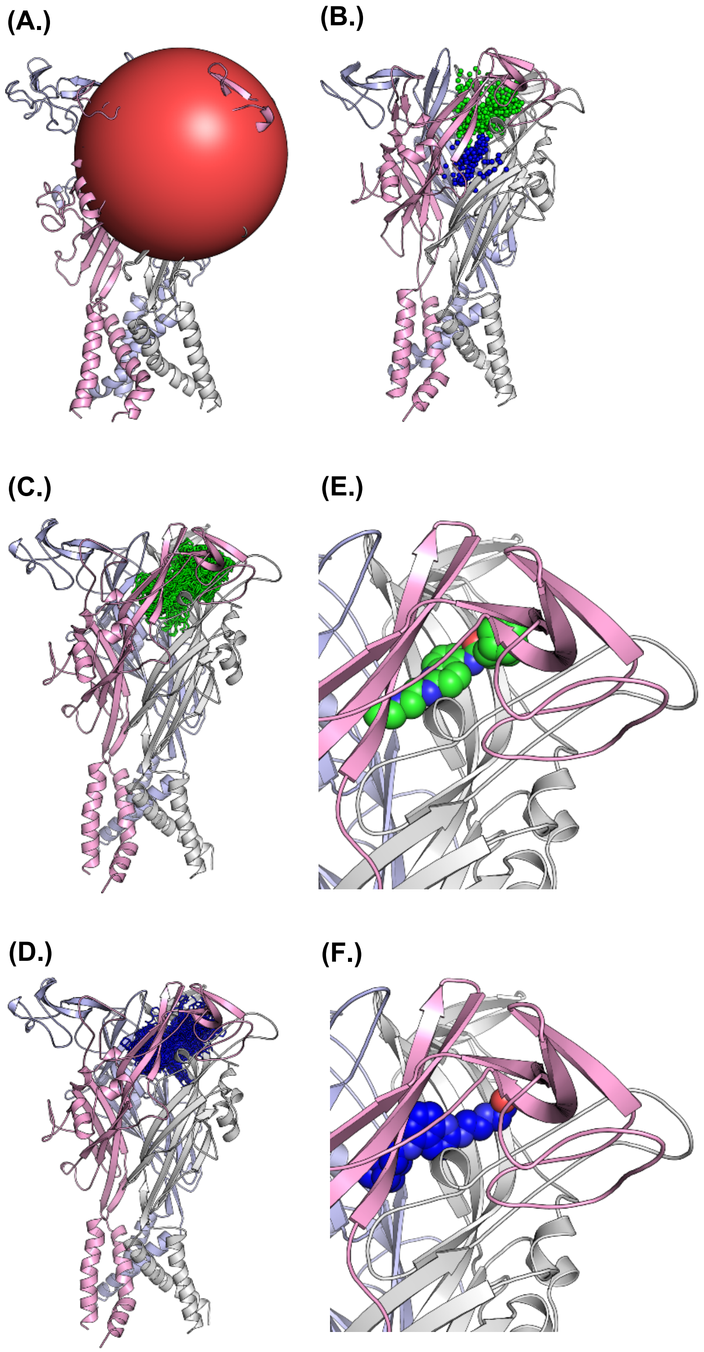

Supplemental Figure 6. Rosetta ligand docking of the AZ10606120. (A.) hP2X7R is shown in cartoon representation with the sampling region (red sphere). (B.) AZ10606120 docking poses covering the orthosteric site (blue) and putative allosteric site (green) shown as spheres representing the centre of mass. (C., D.) Cartoon representation of hP2X7R with the two largest clusters from the second round of focused Rosetta Ligand docking shown as sticks. Cluster 1 (391 poses in green show adamantane group close to the entrance of the putative allosteric pocket and facing “out”) and cluster 2 (365 poses in blue; adamantane group located at the deep end of the pocket facing “in”). (E., F.) Zoom into cluster 1 and cluster 2, respectively. Representative poses from both clusters are shown as sticks.

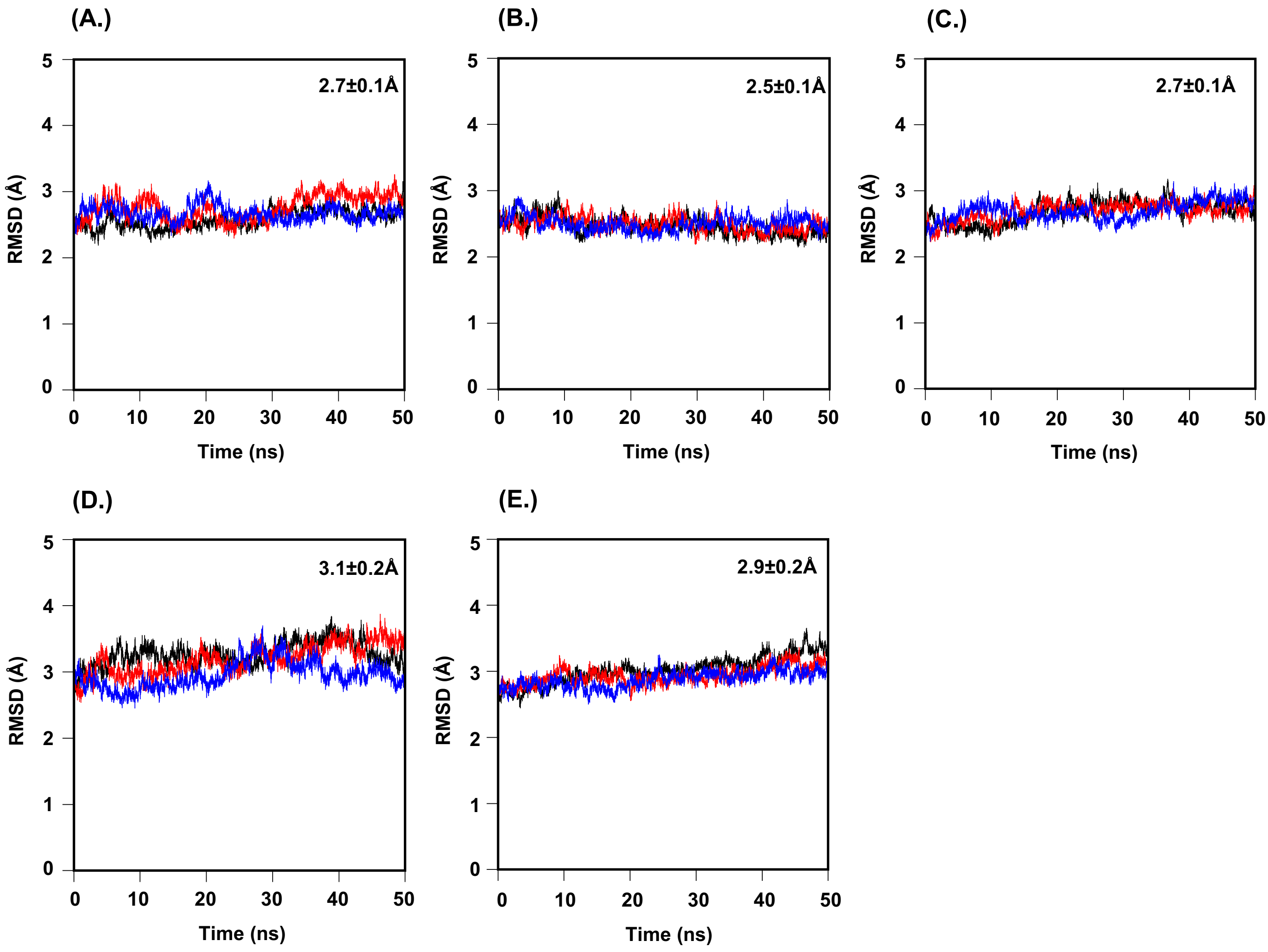

Supplemental Figure 7. Cα-rmsd for molecular dynamics simulations of human P2X7 and P2X1 receptors. (A.) hP2X7R (wildtype). (B.) hP2X7R (deletion mutant 73-79). (C.) hP2X7R (T90V/T94V mutant). (D.) hP2X1R (wildtype). (E.) hP2X7R (open, ATP-bound). Replicate simulations are shown in black, blue and red.

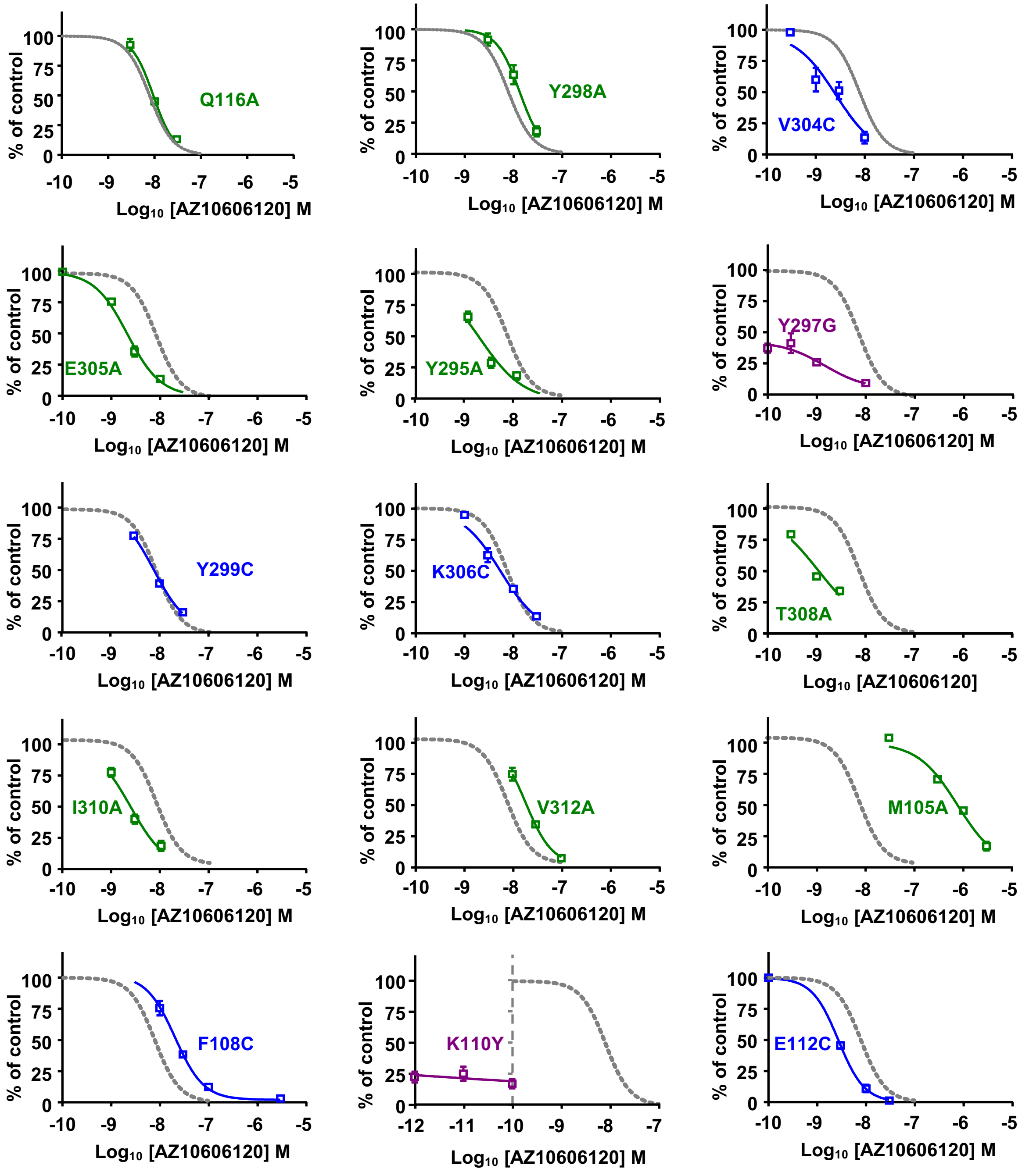

Supplementary Figure 8. Concentration dependence of inhibition of ATP responses by AZ10606120 at point mutants around the allosteric binding site. Histograms show the inhibition (mean ± SEM) of response to an EC_{90} concentration of ATP by AZ10606120. Grey dotted line corresponds to the inhibition at the P2X7-2Nβ receptor. Green corresponds to an alanine mutation, blue cysteine mutation and purple other residue replacement. n= 3-7.
